# Multidimensional study on mitochondrial dysfunction in pulmonary hypertension

**DOI:** 10.3389/fmed.2025.1716485

**Published:** 2025-12-02

**Authors:** Xuntao Yuan, Yutao Zhang, Yuyan Liu, Xiao Guo, Shuying Jia, Xingquan Xiong, Xiuying Sun, Zian Jin

**Affiliations:** 1 Department II of Spleen and Stomach Diseases, Weifang Hospital of Traditional Chinese Medicine, Shandong, China; 2 Department of General Affairs, Weifang Center for Disease Control and Prevention, Shandong, China; 3 Rehabilitation Medicine College, Shandong Second Medical University, Shandong, China; 4 School of Gongli Hospital Medical Technology, University of Shanghai for Science and Technology, Shanghai, China; 5 Department of Pulmonary and Critical Care Medicine, Ruijin Hospital, Shanghai Jiao Tong University School of Medicine, Shanghai, China; 6 Department of Infectious Diseases, Weifang Hospital of Traditional Chinese Medicine, Shandong, China; 7 Cardiac Intervention Center, Eastern Hospital of Weifang Hospital of Traditional Chinese Medicine, Shandong, China

**Keywords:** mitochondrial dysfunction, pulmonary hypertension, oxidative stress, pulmonaryvascular remodeling, research progress

## Abstract

Pulmonary hypertension (PH), as a complex clinical syndrome, can be caused by multiple pathophysiological factors. Its characteristics are similar to hemodynamic abnormalities, significant increase of pulmonary artery pressure, contraction and remodeling of blood vessels, which eventually lead to serious complications such as increased pulmonary vascular resistance, hypertrophy of the right ventricle, and heart failure. The etiology of PH is multifaceted and highly variable, with a common pathological basis primarily characterized by mitochondrial dysfunction. Endothelial cell dysfunction, which directly impacts metabolism and function, is closely associated with PH and other lung diseases, making mitochondrial dysfunction the cornerstone of this condition. The therapy for PH primarily focuses on relaxing pulmonary blood vessels. However, existing vasodilation approaches struggle to effectively reverse the observed vascular remodeling process, which limits further therapeutic enhancement. Moreover, mitochondrial dysfunction represents a promising new direction of significant research in the treatment of PH. This review systematically combs the key molecular mechanisms of mitochondrial dysfunction in the pathological process of PH. The study focuses on multi-channel pathogenic mechanisms, including mitochondrial DNA (mtDNA) damage, electron transfer chain (ETC) dysfunction, protein homeostasis imbalance, defects in mitochondrial biogenesis, dynamic abnormality, and autophagy defect. Furthermore, this review summarizes recent research advancements targeting mitochondrial dysfunction as a potential intervention strategy for clinical treatment of PH. By integrating updated findings on molecular mechanisms with insights from existing literature, the study provides a comprehensive understanding of mitochondrial dysfunction’s role in PH pathogenesis and offers actionable evidence for developing novel therapeutic approaches.

## 1 Introduction

PH is a progressive disease associated with high mortality in both children and adults. The pathological features of PH are endothelial dysfunction, vascular wall hyperplasia, vasoconstriction, inflammatory cell infiltration, and thrombosis (1). At the molecular level, the core feature of vascular remodeling in PH is the excessive proliferation of pulmonary artery smooth muscle cells (PASMC). At the same time, dysfunction of various pulmonary vascular cells, including pulmonary artery endothelial cells (PAEC), jointly drive the pathological process of PH (2). From the perspective of pathophysiological mechanism, the occurrence of abnormal pulmonary vascular system and right ventricular function is closely related to the disorder of various cellular metabolic pathways, including enhanced aerobic glycolysis, pentose phosphate pathway (PPP) activation, abnormal glutamine metabolism and changes in fatty acid oxidation (FAO) process, accompanied by the significant inhibition of glucose oxidation in PVCs, which together promote the pathological process of PH (3). Mitochondrial dysfunction and subsequent oxidative stress are important factors leading to PH diseases (4). In the past 15 years, about 10 vasodilators have been developed for pulmonary vascular endothelial dysfunction involving prostacyclin, nitric oxide-soluble guanylate cyclase-cyclic GMP (NO-sGC-cGMP), and endothelin signaling pathways. Moreover, the latest clinical guidelines emphasize that the combined medication strategy should be adopted according to the severity of the disease, and the severe patients can be treated with triple therapy. However, under the existing treatment scheme, the prognosis of the disease is still not optimistic (5). It is known that mitochondrial dysfunction is an important factor in the pathogenesis of PH. The main purpose of this review is to explain the key changes of mitochondrial dysfunction in PH and provide new insights for developing new PH target drugs.

## 2 Research progress of PH

PH is a clinical syndrome characterized by an abnormal increase of pulmonary artery pressure (6), and it is a rare progressive disease, with high incidence and high mortality, especially in elderly patients over 65 years old (7). The 6th World Symposium on Pulmonary Hypertension (WSPH) defined PH as the average pulmonary artery pressure greater than 20 mmHg, which was characterized by pulmonary vascular contraction and vascular remodeling, resulting in increased pulmonary vascular resistance and abnormal hemodynamic and mechanical functions of pulmonary vessels and right ventricular (RV) posterior (8). According to the high similarity of pathological features, clinical manifestations, hemodynamic indexes, and treatment schemes, PH was divided into five clinical types by WSPH (6), Pulmonary arterial hypertension (PAH), PH associated with left heart disease, PH caused by chronic lung disease or hypoxia, PH caused by chronic thromboembolism, and other types of PH (9). Persistent pulmonary vasoconstriction and excessive occlusive pulmonary vascular remodeling are the main pathological changes of PH formation (10), especially in PAH, which is particularly obvious (11). About 1% of the global population suffers from PH, of which 80% cases are concentrated in developing countries (12). The incidence of idiopathic PAH is 20 cases per million people, and the number of female patients is four times that of male patients (13). PH is mainly pulmonary vascular disease, but RV function directly affects the development of the disease, and the mortality rate caused by right heart failure in PAH patients can exceed 40% (14). It is worth noting that the burden of PH disease faced by low-income and middle-income countries is more severe (15).

Vascular remodeling is a multifactor-driven process involving structural changes such as the apoptosis-resistant and hyperproliferative phenotype of PASMCs, matrix deposition, mitochondrial dysfunction, metabolic disorder, and their underlying molecular mechanisms (16). These changes lead to an imbalance in proliferation and apoptosis signaling pathways, severe disruption of cellular metabolism and metabolic flux, and impaired mitochondrial function (17), while PAECs exhibit increased apoptosis, microvascular loss, and occlusive vascular remodeling (18). Among these, mitochondrial dysfunction has recently emerged as a key pathogenic factor (19). Pulmonary vascular remodeling is characterized by the coexistence of PAEC apoptosis, PASMC hyperproliferation, and extracellular matrix accumulation (16). Pulmonary vascular remodeling involves many pathological mechanisms, including damage to the bone morphogenetic protein receptor 2 (BMPR2) signaling pathway (16), abnormal activation of the growth factor signaling pathway (17), abnormal ion channel function (18), inflammatory injury, oxidative stress (19), abnormal energy metabolism (20), and so on. Mitochondrial dysfunction is related to many mechanisms of pulmonary vascular remodeling, involving all aspects of lung diseases (21). Pulmonary vascular remodeling is driven primarily by sustained proliferation of pulmonary vascular cells and resistance to apoptosis (22), so pulmonary vascular remodeling has been established as the key target of basic research and clinical intervention, which is closely related to the poor prognosis of patients with PH (23).

In the early stage of the disease, hypoxic pulmonary vasoconstriction plays a leading role as a unique physiological reflex mechanism of pulmonary circulation (11). It is a unique physiological reflex of pulmonary circulation, that optimizes the ventilation/perfusion ratio by constricting the arterioles in the hypoventilation area (24). With the progression of the disease course to the middle and late stage, the pathological changes gradually turn into irreversible pulmonary vascular remodeling, and its characteristic changes include four aspects. First, endothelial dysfunction is characterized by the decrease of endothelial nitric oxide (NO) synthase activity, which reduces NO production by 50–70%, while the secretion of endothelin-1 increases by 2–3 times, which leads to the imbalance of vasodilation/contraction factors (25). Second, PASMC proliferate abnormally and their proliferation index is 40–60% higher than normal, accompanied by apoptosis inhibition (26). Third, the specific infiltration of inflammatory cells (27), especially CD4+ T lymphocytes, amplifies the inflammatory response by secreting cytokines such as interleukin-17 (IL-17), and the infiltration density of such inflammatory cells in the lung tissue of patients with PH can reach 3–5 times the normal value (28). Finally, the remodeling of the extracellular matrix leads to an increase in collagen deposition and the rupture of elastic fibers, which eventually form the typical pathological changes of vascular wall thickening (29) and lumen stenosis (30). These structural changes cause a continuous increase in pulmonary vascular resistance. According to clinical guidelines, when the mean pulmonary artery pressure (mPAP) exceeds 25 mmHg, the condition is often classified as entering the overt or clinically dominant stage, distinct from the earlier phase defined solely by an mPAP > 20 mmHg (31).

Currently, Food and Drug Administration (FDA) approved therapies for pulmonary arterial hypertension fall into four classes: nitric oxide-cGMP pathway enhancers, prostacyclin pathway agonists, endothelin receptor antagonists, and sotatercept, an activin-signaling inhibitor that restores BMPR2 signaling (32). These drugs can dilate pulmonary blood vessels and relieve symptoms. But the therapeutic effect is limited, and the 5-year survival rate of patients is only 50–60%. The 5-year survival rate of untreated patients with idiopathic PAH is even lower, only 34% (33). These drugs alone or in combination can significantly improve patients’ functional status, quality of life, hemodynamic indexes, and reduce hospitalization rate. However, even drugs such as intravenous prostaglandin have partial and short-lived effects, and these drugs, which mainly play the role of vasodilation, do not target the core mechanism of the disease (34). The limited efficacy of current PAH therapies primarily stems from their inability to reverse established pulmonary vascular remodeling. Moreover, widely used vasodilators merely ameliorate vasoconstriction without resolving the underlying obstructive vasculopathy or counteracting the cancer-like phenotype of vascular cells. Crucially, most existing strategies do not specifically target the pathologically hypertrophied right ventricle, a key determinant of disease progression and mortality (35).

The initial PH research focused on hypoxia and hypoxic pulmonary vasoconstriction (HPV) mechanism (36). Although the specific mechanism of HPV is unknown, it is suggested that mitochondria promote reactive oxygen species (ROS) production through ETC, which may mediate this process. Subsequent studies have found that mitochondrial dysfunction plays a more extensive role in PH, and it may be involved in the occurrence of pulmonary vascular diseases even without hypoxia (37). Recent research results further reveal that mitochondrial dysfunction and metabolic imbalance are also the key incentives for pulmonary vascular remodeling. It is of great academic value to explore the molecular mechanism of vascular remodeling, especially the role of mitochondrial dysfunction (38). Targeting mitochondrial dysfunction as a therapeutic strategy for PH represents a critical and urgent challenge.

## 3 Mitochondrial dysfunction in PH

### 3.1 The normal mitochondria

Mitochondria are highly dynamic double-membrane organelles, including the outer mitochondrial membrane (OMM) and inner membrane, which separate the space between membranes and matrix (39). Mitochondria are the most important energy-producing parts of the human body, and are known as recognized oxygen and fuel sensors (40). The inner mitochondrial membrane (IMM) is the core of protein transport and oxidative phosphorylation (OXPHOS). Respiratory chain complex I-IV and Adenosine triphosphate (ATP) synthase operate on it, driving electron transfer and proton-motive force (Δp), and supporting ATP synthesis. Acetyl-CoA and α-ketoglutarate (α-KG) feed into the tricarboxylic acid (TCA) cycle, generating NADH and FADH2. These reduced cofactors donate electrons to the respiratory chain complexes, which pump protons into the intermembrane space and establish a proton-motive force. Finally, ATP synthase utilizes this electrochemical gradient to synthesize ATP, powering cellular processes (41).

Mitochondria, which originated from bacteria, retain unique mtDNA and encode the key components of the respiratory complex. The mitochondrial respiratory chain has the characteristics of a double genome, and its 13 core proteins are encoded by mtDNA, which contains 24 genes encoding RNAs at the same time. However, most respiratory chain-related proteins are encoded by nuclear genes. The normal function of mitochondria depends on the co-expression of the nuclear genome and mitochondrial genome, and any gene mutation can lead to mitochondrial dysfunction (42). In recent years, it has been found that mitochondria can be transmitted between cells through a non-hereditary horizontal transfer mechanism (43). Healthy mitochondria can repair the energy metabolism function of recipient cells (44), while damaged mitochondria maintain tissue homeostasis through a macrophage-mediated clearance mechanism (45). More than 90% of cellular ATP is generated through OXPHOS (46).

Mitochondria, as the energy factory and metabolic control center of eukaryotic cells, are regulated by a multi-level quality control mechanism. Their functions are mainly reflected in three aspects: quality control, energy generation, and metabolic control (47). As a pleiotropic organelle, it orchestrates a spectrum of vital cellular events, including Ca^2+^ homeostasis (48), ROS generation (49), apoptosis, oxidative stress buffering, signal transduction, and lipid/heme biosynthesis (50).

### 3.2 Mitochondrial dysfunction

The concept of mitochondrial dysfunction originated in the context of bioenergetics and has since expanded to encompass interactions with the cellular microenvironment. Mitochondrial dysfunction originally originated from bioenergy, and now it has extended to the relationship between the cell environment (51). Mitochondrial-related bioactive molecules, such as mtDNA, mitochondria-located microRNA, and various functional proteins, have potential therapeutic value in improving mitochondrial function in the process of immune metabolic diseases and tissue injury repair (52). Mitochondrial abnormalities are not only seen in primary diseases, but also widely involved in secondary pathological processes. Primary mitochondrial diseases are caused by mtDNA defects, and mtDNA heterogeneity accumulates with aging, which aggravates clinical manifestations. Secondary mitochondrial dysfunction (SMD) is common in heart failure, neurodegenerative diseases, etc., where abnormal dynamics, protein homeostasis, and other factors work together to affect mitochondrial function. It is worth noting that in the context of PH, the mutation, deletion, and abnormal replication of mtDNA are regarded as the key factors leading to mitochondrial dysfunction in molecular pathological mechanisms, triggering a cascade reaction. These pathological changes further aggravate the progression of PH through a series of cascade effects.

Mitochondrial dysfunction is closely related to many pathological mechanisms of pulmonary vascular remodeling. It mainly participates in the characteristic vascular remodeling of PH through the following mechanisms: firstly, dysfunction of PAEC leads to imbalance of vascular tone regulation and abnormal anti-proliferation signal conduction; secondly, PASMC proliferate abnormally; thirdly, metabolic reprograming, characterized by a shift from oxidative phosphorylation to glycolysis akin to the Warburg effect in cancer, provides energy support and biosynthetic raw materials for cell proliferation; in addition, the activation of signal pathways induced by oxidative stress promotes the excessive deposition of extracellular matrix; finally, ROS-mediated release of inflammatory factors and infiltration of inflammatory cells aggravate vascular inflammatory reaction.

Mitochondria, as the energy center of cells, is very important to maintain their redox balance. Mitochondrial dysfunction will not only lead to energy metabolism disorder of PASMCs, but also produce a large number of ROS, which will increase oxidative stress and activate an inflammatory response (48). Mitochondrial dysfunction can promote abnormal proliferation of vascular cells, inhibit apoptosis, and drive pulmonary vascular remodeling through mechanisms such as ROS accumulation and metabolic disorder (37), and any functional imbalance can affect the process of vascular remodeling (49). Studies have shown that mitochondrial dysfunction and its Warburg effect significantly affect the pulmonary vascular remodeling process in the PAH field (50). Optimizing the glucose oxidation pathway, repairing mitochondrial damage, inhibiting mitochondrial division and autophagy, and regulating mitochondrial calcium homeostasis may become a potential experimental treatment for PAH (53).

**FIGURE 1:**
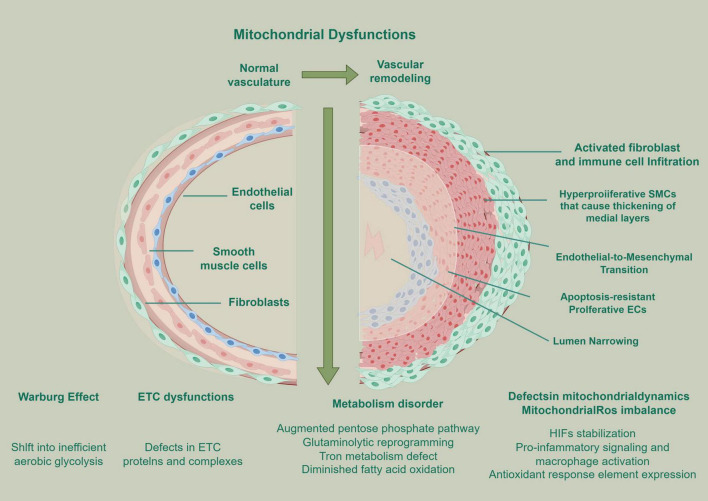
Mitochondrial dysfunction and its role in pulmonary vascular remodeling, aiming at the vascular remodeling mechanism of PH. Created by Figdraw.

From animal models to clinical studies, it has been confirmed that there is a significant correlation between the abnormality and dysfunction of mitochondrial structure and the formation and progression of PH. This relationship is mainly manifested in the imbalance of the mitochondrial quality monitoring system, which covers the obstacles of key processes such as mitochondrial production, dynamic balance of fusion and division, and mitochondrial autophagy (53). These abnormal states are involved in the process of pulmonary vascular remodeling through complex pathological mechanisms (54). Mitochondrial dysfunction related to PH is mainly reflected in the following aspects, which constitute its core features. First, dysfunction of ETC, imbalance of ETC-related protein expression level, and significant changes in enzyme activity, which lead to the reduction of OXPHOS efficiency (48). Second, the remarkable imbalance of mitochondrial dynamics has broken the balance of mitochondrial fusion and division, which has seriously affected the normal morphology and function of the mitochondrial network (55). Third, the destruction of ROS and the dysfunction of ETC function will induce the excessive production of ROS, which will further aggravate the oxidative stress (56). In addition, with the reprogramming of metabolic pathways, the metabolic mode of cells has changed from OXPHOS to glycolysis, that is, the Warburg effect, to meet the energy demand (57). Abnormal regulation mechanism of apoptosis and autophagy leads to abnormal proliferation or survival of vascular cells (58). Finally, Damage to the mitochondrial respiratory chain can decouple the ETC from ATP, induce ROS production, and lead to mitochondrial dysfunction (59). Superoxide dismutase 2 (SOD2) and Superoxide dismutase 1 (SOD1) participate in the transformation of hydrogen peroxide (H_2_O_2_) (60), which is an important signal molecule of ROS (61). Mitochondrial ROS is regulated by enzymes, ETC, and electrochemical barriers, which can induce apoptosis (62). ATP synthesis disorder may lead to multi-level physiological mitochondrial dysfunction (63). When any of the above functions is disordered, it indicates that mitochondrial function is abnormal (64) (Figure 1).

## 4 mtDNA damage

When the accumulation of mtDNA mutations in cells exceeds the threshold of 60–80% heterogeneity, it will lead to the synthesis of defective ETC components, which will lead to mitochondrial dysfunction and phenotypic expression (65). Mitochondrial dysfunction leads to the release of mtDNA in the form of DAMP (66). By activating immune pathways such as toll-like receptor 4 (TLR4) and NOD-, LRR- and pyrin domain-containing protein 3 (NLRP3) inflammatory corpuscles, it triggers an inflammatory reaction and promotes the development of PH (67), thus forming a vicious circle of mitochondrial injury, inflammation, and histopathology. mtDNA is more vulnerable to oxidative damage than nuclear DNA, especially when mitochondria produce ROS (68). The extent of injury is tightly linked to the abundance of mitochondrial oxidative-repair enzymes. When these enzymes are depleted cytotoxicity and apoptosis are amplified, whereas their overexpression confers protectio (69). Oxidative damage of mtDNA will further lead to mitochondrial dysfunction and apoptosis, but this process can be partially alleviated by 8-oxoguanine DNA glycosylase (Ogg1) (70). Ogg1 plays a key role in this process, which can not only alleviate mitochondrial dysfunction and apoptosis caused by oxidative damage of mtDNA, but also protect against lung injury induced by ventilator and hyperoxia (71), and also plays an active role in the prevention and treatment of PH (72).

In the process of apoptosis, mtDNA will be released in fragments, which can be used as a damage-related molecular pattern to activate the innate immune response (69). These fragments act through two main pathways, TLR4 and NOD-like receptor, such as the inflammasome of NLRP3 (73). On the one hand, the activation of TLR4 is related to PH occurrence, and it is closely related to the development of vascular diseases in sickle cell disease (67). The activation of toll-like receptor 9 (TLR9) may form a feed-forward cycle and aggravate the damage of mtDNA (74). The activation of inflammatory corpuscles of NLRP3 is involved in the pathogenesis of PH, and animal experiments show that inhibiting this pathway can prevent the progression of PH. In addition, oxidative stress caused by hypoxia/reoxygenation can not only damage mtDNA but also enhance the activity of caspase-3/7 in PAEC, thus affecting the reversibility of PH (75).

## 5 ETC dysfunction

In PH, the dysfunction of the mitochondrial respiratory chain is related to glycolysis transfer (76). Studies show that glycolytic enzyme α-enolase (ENO1) is involved in the metabolic reprograming of PASMC, and its expression is increased in patients with PH and animal models. Inhibition of ENO1 can reduce the proliferation and induce apoptosis of PASMC, while overexpression can promote the dedifferentiation and apoptosis resistance of PASMC through the AMPK-Akt pathway (77). Mitochondrial dysfunction plays a central role in the pathogenesis of PH. Studies have shown that ETC-deficient mitochondria are characterized by respiratory chain decoupling and decreased oxygen utilization efficiency (78), which are closely related to chronic inflammatory reaction (79) and abnormal proliferation of vascular PASMC in the course of PH (80). Mitochondria act as a central signaling hub that generates α-KG and ROS to modulate transcription factors such as HIF-1α and nuclear factor of activated T-cells (NFAT), thereby driving vascular remodeling. In addition, mitochondrial metabolites, such as α-KG and citrate, can affect the development of PH by regulating epigenetic modifications such as histone methylation/acetylation (81). Studies have confirmed that the mitochondrial apoptosis pathway is markedly suppressed in PH (40). It is worth noting that mitochondrial dysfunction can activate NLRP3 inflammatory corpuscles, leading to an increase in inflammatory factors and forming a vicious circle (81). These findings jointly established the central position of mitochondria in the pathological process of PH.

As the key organelle of energy metabolism in eukaryotic cells, mitochondria will continuously produce a large number of free radical molecules such as ROS and reactive nitrogen species (RNS) through the TCA cycle and other physiological activities (82). ETC not only synthesizes ATP, but also maintains the balance of mitochondrial membrane potential in the process of OXPHOS (83). When the mitochondrial function is impaired, it can promote the outflow of cytochrome C, and then activate the apoptosis signal pathway (84). It is worth noting that in addition to the productivity function, the metabolic intermediates of the TCA cycle, such as acetyl-CoA (85), α-KG acid (86), and fumaric acid (87), have important signal transduction functions. In anoxic microenvironments, ETC complexes I and III become the main production sites of superoxide anion (O^2–^) and H_2_O_2_, and their contribution can reach more than 80% of the total ROS in cells (88). In addition, the NADPH oxidase system, ETC, together constitute two enzymatic pathways of ROS production in cells, and their dysfunction is closely related to mitochondrial dysfunction (89).

## 6 Mitochondrial quality control

**FIGURE 2:**
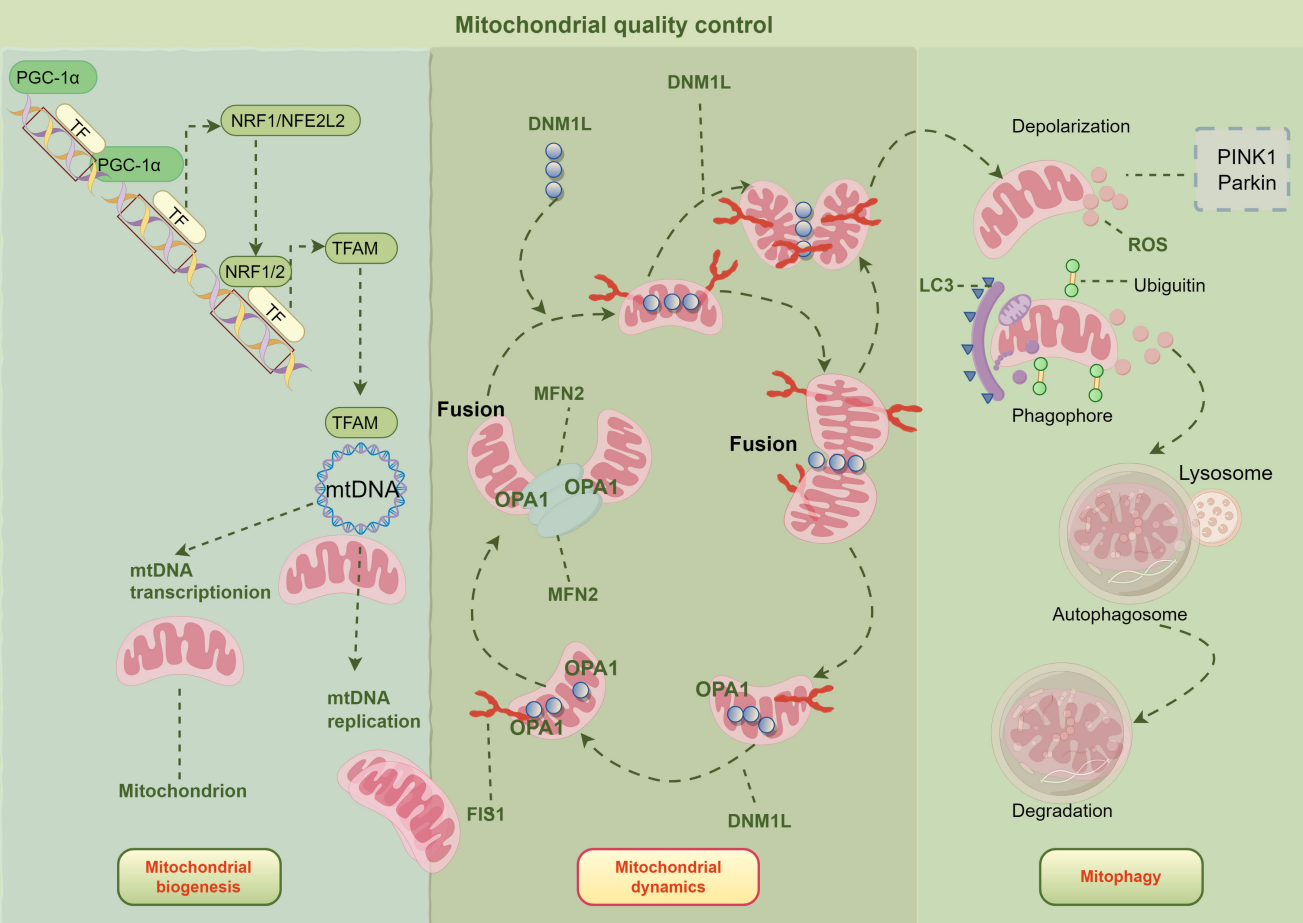
Mitochondrial quality control approach. Created by Figdraw.

Mitochondrial quality control is the key mechanism to maintain mitochondrial health and cell bioenergy. These processes include protein homeostasis, biogenesis, kinetics, and mitochondrial autophagy (90) (Figure 2). The regulation mechanism of mitochondrial homeostasis involves biosynthesis, dynamic equilibrium, and selective autophagy. Mitochondrial homeostasis is mediated by peroxisome proliferator-activated receptor-γ coactivator-1α/nuclear respiratory factor-1 (NRF1)/mitochondrial transcription factor A (TFAM) pathway (91). In biosynthesis, peroxisome proliferator-activated receptor gamma coactivator 1-alpha (PGC-1α) regulates the transcription and replication of mtDNA by activating nuclear transcription factors such as NRF1, nuclear factor erythroid 2-related factor 2 (NRF2), and estrogen-related receptor-α (ERR-α) (92). Dynamin-related protein 1 (DRP1) regulates the division process mediated by its binding with mitochondrial fission 1 (FIS1) and other receptors through Ser site-specific phosphorylation, while mitofusin 1 (MFN1), mitofusin 2 (MFN2), and optical atrophy 1 (OPA1) are responsible for the fusion of the OMM and inner membrane, respectively. In selective mitophagy, the PTEN-induced kinase 1 (PINK1)–Parkin pathway ubiquitinates mitochondrial outer-membrane proteins, thereby recruiting the autophagy receptor CALCOCO2/NDP52 and initiating autophagosomal engulfment of damaged mitochondria (93). TFAM can increase ATP production by 30–50% by enhancing the transcription of the ETC gene, while abnormal mitochondria are selectively removed after ubiquitination labeling. In the PH model, a series of interruptions of mitochondrial quality control were observed (94). The process of mitochondrial quality control ensures healthy mitochondrial function and cellular bioenergy.

### 6.1 Imbalance of mitochondrial protein homeostasis

The imbalance between mitochondria and protein will lead to abnormal mitochondrial function (95). The maintenance of mitochondrial homeostasis needs to be achieved through multiple quality control mechanisms, which are embodied in key biological processes such as the regulation of mitochondrial dynamics, the generation of new mitochondria, and the elimination of dysfunctional mitochondria through selective autophagy (96). Mitochondria are the core organelles of cell energy metabolism, and their protein homeostasis plays a key role in maintaining pulmonary vascular homeostasis. PAEC, PASMC, mesenchymal cells, and immune cells in the pulmonary vascular system all showed significant mitochondrial dynamic changes (54).

Mitochondrial protein homeostasis maintains functional integrity through multi-level regulatory mechanisms. In terms of protein quality control, molecular chaperones like heat shock protein 60 (HSP60) and heat shock protein 70 (HSP70) ensure correct protein folding, while Lon protease 1 (LonP1) mediates the degradation of abnormal proteins. Furthermore, the Translocase of the outer mitochondrial membrane (TOM) and translocase of the Inner mitochondrial membrane (TIM) complexes strictly regulate the transmembrane transport of proteins. Together, these systems ensure the normal function of the approximately 1,200 proteins that form the mitochondrial proteome (97). In terms of energy metabolism, by stabilizing the ETC complex and TCA cycle enzyme system, the efficiency of ATP supply in cells can be maintained as high as 90%. It is worth noting that compared with the research depth of endoplasmic reticulum unfolded protein reaction (UPR), there is still a significant cognitive gap in the molecular mechanism of misfolded mitochondrial proteins in the pulmonary circulation system (98). From the point of view of experimental basis, mitochondrial protein homeostasis plays a key role in the pulmonary circulatory system and the pathological process of PH. It was found that the lung tissue of rats treated with mitochondrion complex III inhibitor antimycin A showed obvious dysfunction of protein clearance and detoxification, which may be an important mechanism leading to pulmonary vasoconstriction. This phenomenon directly confirms the causal relationship between mitochondrial protein homeostasis disorder and pulmonary circulatory dysfunction (99). The abnormal expression of proteases such as Lon protease 1 (LonP1) in PAEC of PAH patients is positively correlated with the accumulation of mitochondrial false proteins and the degree of vascular remodeling. From the perspective of pathological mechanisms, in the pathological process of PH, the imbalance of mitochondrial protein homeostasis may affect the development of the disease through multiple mechanisms. Notably, the observed cardioprotective mechanism in myocardial ischemia-reperfusion injury, which involves the upregulation of mitochondrial protease expression, suggests that enhancing mitochondrial protein quality control could be a promising therapeutic strategy to alleviate PH-associated RV dysfunction, thereby identifying a potential pathway for clinical intervention (100).

### 6.2 Mitochondrial dynamic abnormality

Mitochondrial dynamics refers to the delicate balance maintained between the two dynamic processes of mitochondrion division and fusion (101), and their division and fusion processes are regulated by the localization of the skeletal system (102). This dynamic mechanism plays a key role in maintaining cell cycle, proliferation, apoptosis, and quality control by regulating the morphology, distribution, and volume of mitochondria (103). The dynamic changes between mitochondria are regulated by the signal transmission process between OMM and mitochondria-related membranes (51). Abnormal mitochondrial dynamics lead to mitochondrial dysfunction, accompanied by a high concentration of ROS, which has become a key indicator of many lung diseases in the early stage (104). Mitochondria can fission or fuse flexibly according to the needs of cells, so as to optimize their functions and maintain an appropriate number and distribution, thus further enhancing the quality control function of mitochondria (90).

Mitochondrial fusion is mediated by large Guanosine TriPhosphatases (GTPases) at both membranes: MFN1/2 on the outer membrane and OPA1 on the inner membrane. MFN1/2 are primarily responsible for outer membrane fusion. This process is crucial for enhancing mitochondrial functional capacity and optimizing the efficiency of ATP production (105). Mitochondrial fusion aims to optimize the work of mitochondria by transferring gene products, while mitochondrial fission aims to maintain their proper number and distribution (90). Once this equilibrium state is destroyed, it may cause many diseases, including PH. The dynamic regulation of mitochondrial morphology mainly depends on fission and fusion-related proteins, among which mitochondrial fusion proteins such as MFN1/2 and OPA1 protein constitute the core molecular mechanism of mitochondrial membrane fusion (106). Mitochondrial fusion will lead to the remarkable restructuring of two organelles. This dynamic event involves the integration of four lipid membranes, which not only realizes the redistribution of membrane components but also promotes the mutual fusion of mitochondrial matrix contents. Studies have shown that when cells lack mitochondrial fusion protein or OPA1, it will lead to multiple dysfunctions, which are manifested as mitochondrial membrane potential disorder, abnormal nuclear-like structure of mtDNA, and significant decline in OXPHOS efficiency (107). Experimental genetic evidence shows that Mfns family proteins and OPA1 have a clear division of membrane fusion according to their subcellular localization, OMM fusion is mediated by Mfns, while inner membrane fusion depends on the GTPase activity of OPA1, and they cooperate to realize the dynamic remodeling of mitochondrial reticular structure (106).

By contrast, mitochondrial division is primarily driven by DRP1 together with dynamin-2 (DNM2) (108). DRP1 docks to outer-membrane receptors FIS1 and MiD49/51, oligomerizes into a spiral that tightens into a constrictive ring, andnDRP1 s, while inner membrane fusio (109). The initiation of the mitochondrial fission process depends on the directional transport of cytoplasmic DRP1 protein to the mitochondrial membrane, which requires membrane ankyrin, such as FIS1 and mitochondrial fission factor (MFF), as receptor complexes to participate in synergistic regulation (110). The research shows that the MiD family protein and DRP1 form a dynamic interaction network; the former acts as a membrane positioning scaffold to form a ring-shaped lesion, and the latter acts as an effector molecule to perform membrane contraction function, and the two cooperate to ensure the temporal and spatial accuracy of mitotic events (111).

In patients with PAH, mitochondrial dynamic imbalance directly leads to abnormal proliferation and vascular remodeling of PASMC (112). As a commonly used drug in the clinical treatment of PAH, treprostinil (113) has been proven to promote phosphorylation of DRP1 through a protein kinase A (PKA)-dependent pathway. This post-translational modification can effectively inhibit the activity of DRP1 and then enhance the process of mitochondrial fusion in PASMC, which is manifested by the remarkable extension of the mitochondrial network (114). In PAH, overexpression of DRP1 leads to over-proliferation, while treprostinil can promote DRP1 phosphorylation by PKA and increase mitochondrial fusion. Box-1 (HMGB1) in the high mobility group leads to phosphorylation and fission of DRP1 by activating the extracellular signal-regulated kinase 1 and 2 (ERK1/2) pathway and autophagy (115). Mitochondrial fusion is regulated by MFN1, MFN2, and OPA1. MFN2 has an anti-proliferation effect, and its downregulation leads to mitochondrial fragmentation and cell proliferation (116). Transcriptional cofactors such as PGC-1α and ERR-α, and stimulation such as endothelin-1 and PDGF, will affect the expression of MFN2 (117). The role of MFN1 in PH has not been fully understood, but the activation of OPA1 can promote mitochondrial fusion (118).

### 6.3 Defects in mitochondrial biogenesis

Studies have shown that mitochondrial biogenesis is the process by which cells generate new mitochondria to ensure and increase the mitochondrial population (119). The patients with PH showed obvious dysfunction of mitochondrial biogenesis, which was manifested by the decrease of mitochondrial number, morphological abnormality, and the decrease of mtDNA replication and transcription efficiency. This defect further led to the decrease of mitochondrial respiratory chain complex activity, insufficient ATP synthesis, and excessive accumulation of ROS (120). Mitochondrial biogenesis is regulated by nuclear and mitochondrial genomes (81). Mitochondrial characteristics are rapidly regulated by transcription factors such as NRF1, NRF2, and TFAM (121). In the process of mitochondrial biogenesis, cells regulate mtDNA synthesis through PGC-1α and TFAM to increase the number of mitochondria (122). PGC-1α, as a key regulator of mitochondrial biogenesis, integrates upstream signals and activates downstream gene programs (123). It promotes TFAM expression by regulating NRF1/2 (124). And then drives mitochondrial production. After an external inducer activates PGC-1α, it can stimulate NRF and increase TFAM expression (122).

In the pathogenesis of PH, many signaling pathways regulate mitochondrial biogenesis. Studies have shown that NO/cGMP and HO-1/CO signaling pathways play a key role in it (125). The experiment confirmed that NO donor could reverse the expression of PGC1α, ETC complex, and the decrease of mtDNA copy number in the fetal sheep model with PH (48). NO and its derivatives promote mitochondrial biogenesis and are being used as PH therapy in clinical trials (126). HO-1 agonist can prevent the occurrence of hypoxic PH and inhibit pulmonary vascular remodeling (127). It is worth noting that SIRT family proteins play an important role in maintaining mitochondrial function by regulating the post-translational modification of PGC-1α (128). Sirtuin 3 (SIRT3) deletion will significantly affect the expression of mitochondrial coding genes and nuclear coding genes. Clinical and animal experiments have found that SIRT3 functional deletion polymorphism is closely related to PH (129). In addition, Sirtuin 1 (SIRT1)/SIRT3 also influences the mitochondrial membrane potential and function by regulating the deacetylation process of cyclophilin D. These findings provide a new molecular target for the treatment of PH (73). Pharmacological activation of mitochondrial biogenesis can enhance oxidative metabolism and tissue bioenergy, and improve organ function characterized by mitochondrial dysfunction (130). AMPK, as a key regulator of energy metabolism, promotes mitochondrial synthesis in multiple ways to cope with the energy crisis (131). Studies have shown that β-Guanidinopropionic acid (β-GPA) can continuously activate AMPK in skeletal muscle by reducing the ATP/AMP ratio, and long-term intervention can significantly improve NRF-1 activity and mitochondrial-related protein expression (108). Gene knockout experiments confirmed that AMPK deletion could completely block the mitochondrial proliferation induced by β-GPA and inhibit the expression of key factors such as PGC-1α (109). Given diseases related to mitochondrial dysfunction, future research may be aimed at the molecular mechanisms regulating mitochondrial biogenesis and function, and finding new therapeutic targets (94).

### 6.4 Mitochondrial autophagy defect

Mitochondria, as an energy factory of cells, are easily damaged by active intermediates, so they need to be constantly updated to maintain their normal operation (132). When cells face oxidative stress, the steady state of mitochondrial proteins will be destroyed, which may accelerate the process of cell death (133). In order to meet this challenge, cells have evolved a quality control mechanism called mitochondrial autophagy, which can selectively degrade damaged mitochondria, thus helping cells restore internal balance after stress (134). Mitochondrial autophagy is an evolutionarily highly conservative selective autophagy process, which was first systematically expounded by Lemasters’s team in 2005 (135). As an important quality control mechanism in cells, its main function is to selectively identify and remove abnormal or redundant mitochondria through double-layer membrane autophagy, and then fuse with lysosomes to complete degradation (134). This process plays a key role in maintaining the balance of energy metabolism in cells (136) and can contribute 30% of ATP supply in cells under the condition of nutrient deficiency (137).

Mitochondrial autophagy can be divided into non-selective and selective types, which involve different mitochondrial processing mechanisms (138). PINK1/Parkin-mediated mitochondrial autophagy is the core mechanism to maintain mitochondrial homeostasis, and its regulation process has the characteristics of precise dynamic equilibrium (139). In a physiological state, healthy mitochondria degrade PINK1 in IMM by presenilin-associated rhomboid-like (PARL) protease to maintain the basic level. When mitochondrial damage leads to a decrease in membrane potential, PINK1 accumulates in the OMM of mitochondria and is activated by autophosphorylation, thereby phosphorylating ubiquitin and recruiting Parkin ubiquitin ligase. Parkin-mediated ubiquitination of OMM protein combines with microtubule-associated protein 1A/1B-light chain 3 (LC3) to form autophagy, and finally selectively removes damaged mitochondria through the lysosomal pathway, while normal mitochondrial fragments can be repaired through fusion (140).

Mitochondrial autophagy produces fragments with different membrane potentials through DRP1-mediated fission. High-potential fragments realize mitochondrial network reconstruction through fusion protein MFN1/2, while low-potential fragments activate the PINK1/Parkin pathway (141). This selective clearance mechanism can eliminate the dysfunctional mitochondria caused by oxidative stress, and at the same time, keep healthy fragments to complete biosynthesis through OPA1-mediated fusion (142), forming a fission-autophagy-fusion dynamic equilibrium system (143). Mitochondrial autophagy, fission, and fusion jointly maintain mitochondrial homeostasis. Mitochondrial autophagy is divided into membrane potential fragments by DRP1, and the high potential fragments are fused with MFN1/2 to reconstruct the mitochondrial network, while the low potential fragments activate the PINK1/Parkin pathway to remove the damaged fragments (144). Although the specific role of mitochondrial autophagy in PH has not been fully clarified, existing studies have shown that it may be closely related to the development of PH and other lung diseases (145). In PASMC, the increase of mitochondrial autophagy seems to be related to cell proliferation, while some molecules, such as UCP2, can regulate the process of mitochondrial autophagy (146). However, the exact role of mitochondrial autophagy in PAH patients is still controversial. These different conclusions may suggest that there is some correlation between the imbalance of mitochondrial autophagy and the development of PH (38). Therefore, it is of great significance to further study the mechanism of mitochondrial autophagy in PH to understand the pathological process of the disease and develop new therapeutic strategies.

## 7 Therapeutic target of mitochondrial dysfunction in PH

**FIGURE 3:**
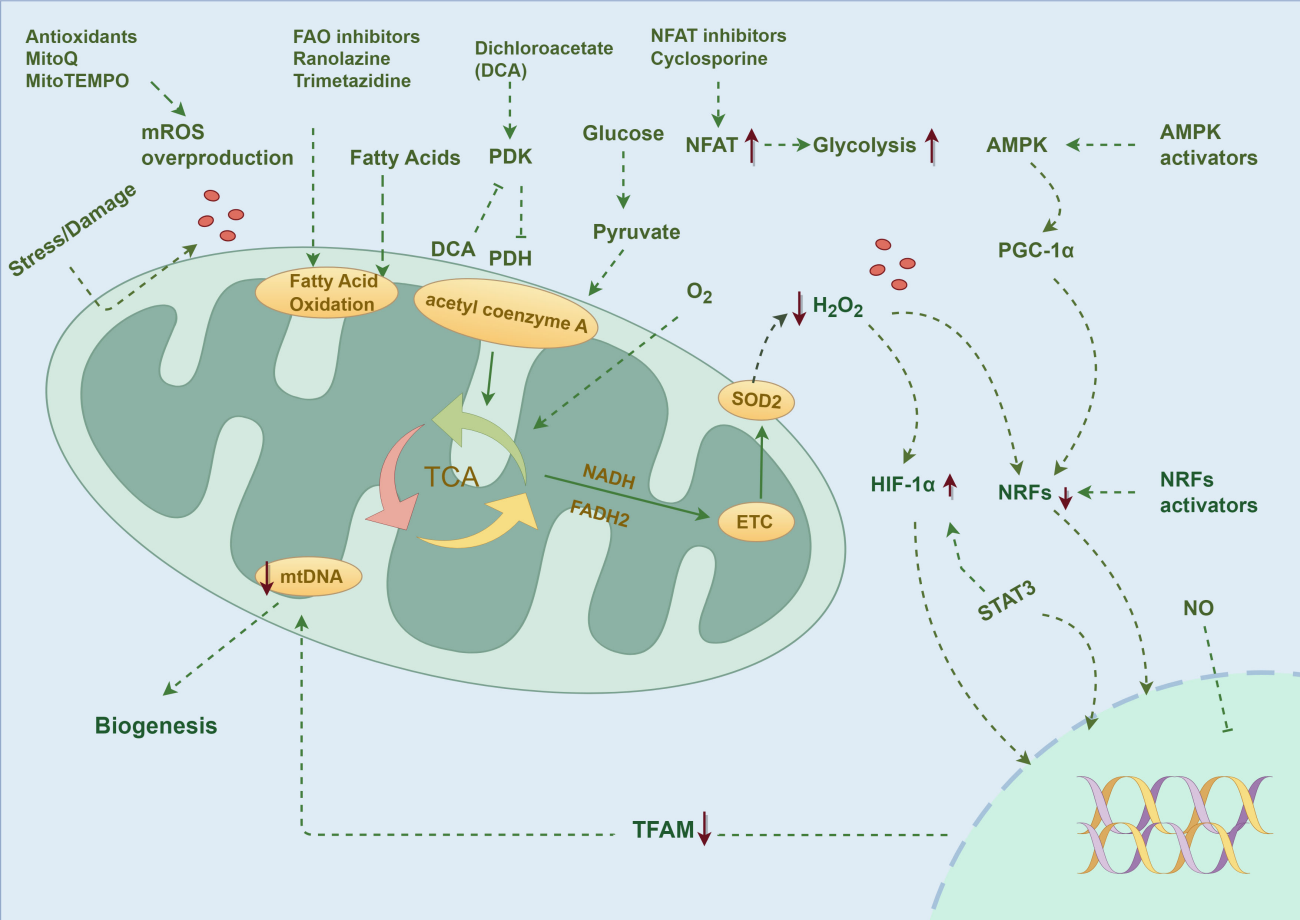
PH is accompanied by mitochondrial dysfunction, and the research focuses on molecular targeted therapy, drug therapy, regulation of metabolic pathways, and transcription factors. Created by Figdraw.

The latest research progress in the treatment of PH shows that mitochondrial dysfunction has become a key therapeutic target. At present, the therapeutic strategies for mitochondria mainly focus on the following directions (Figure 3).

### 7.1 Treatment of mitochondrial targeted drugs

Mitochondrial-targeted drugs provide a new idea for PH therapy. Aiming at the key target of mitochondrial metabolism, the therapeutic strategy of regulating the balance between glycolysis and OXPHOS shows good prospects. At present, a variety of mitochondrial targeting drugs have entered clinical research drugs targeting glucose metabolism, such as dichloroacetate (DCA), can reverse PAH and Warburg effects in mice *in vitro* (147), Glycolytic pathway was enhanced, and related proteins such as HIF-1α, HIF-2α and pyruvate dehydrogenase kinase (PDK) were up-regulated (48), The drug enhances pyruvate dehydrogenase (PDH) activity by inhibiting PDK2, and promotes energy metabolism to aerobic respiration instead of glycolysis, and can also regulate hyperproliferation and oxidative stress through p38 signaling pathway when combined with atorvastatin (148). The human trial of DCA initially confirmed its potential to improve hemodynamics in patients with PAH, and the animal model also showed that DCA could reverse pulmonary vascular remodeling induced by hypoxia (149). In addition, inhibitors of PFKFB3 and ENO1 have also demonstrated efficacy in suppressing the development of PAH in animal models (150).

Since mitochondrial dysfunction will lead to a decrease in glucose oxidation utilization rate and an increase in FAO, researchers can try to use FAO inhibitors, including trimetazidine and ranolazine, to reverse the above metabolic abnormalities (20). Ranolazine is an FAO inhibitor, which indirectly activates PDH by lowering the level of acetyl coenzyme A. It showed a significant protective effect in the MCD-deficient mice model. Clinical studies showed that although mPAP was not improved (151). The PH mechanism involves cell proliferation and mitochondrial division. Another drug of FAO is trimetazidine, which can inhibit the proliferation and metabolic transformation of PASMC. Revealing the molecular pathway of metabolic disorder caused by mitochondrial morphological changes may be helpful to the treatment of pulmonary vascular diseases (152). Trimetazidine has been approved as an anti-angina drug and has shown remarkable efficacy in animal models of PH induced by chronic hypoxia and monocrotaline, and its clinical verification is currently in the experimental stage. The drug plays a role through a unique metabolic regulation mechanism, which can maintain cell energy metabolism under ischemia and hypoxia, prevent ATP levels from falling, and ensure the normal function of the ion pump. Animal studies show that trimetazidine can help maintain the energy metabolism of the heart and nerve sensory organs under hypoxia, reduce intracellular acidosis, and reduce neutrophil infiltration during ischemia, and has no obvious effect on hemodynamics. At present, the clinical evidence of its treatment of PH is still accumulating, and the latest research shows that the drug may play a therapeutic role by protecting mitochondrial function (153). As a calcineurin inhibitor, cyclosporine A (CsA) suppresses abnormal PASMC proliferation by blocking NFAT nuclear translocation. In monocrotaline-induced rat models of PAH, CsA reduces mean pulmonary arterial pressure by approximately 25% and ameliorates vascular remodeling by 30%. However, clinical trials reveal significant individual variations in its efficacy and side effects such as renal impairment, necessitating further validation in large-scale Phase III trials (154). These examples show that although the therapeutic strategy for mitochondria is attractive in theory, there are still many challenges to be overcome in practical application. Although there are more and more treatments for PH, the effect of single therapy on improving the prognosis of patients is limited. At present, a multi-target combined therapy strategy is recommended (155). However, the latest study found that macitentan combined with tadalafil and selepag failed to significantly improve pulmonary vascular resistance (156), suggesting that a larger-scale randomized trial is needed to evaluate the difference in therapeutic effects of different combined drug regimens.

### 7.2 Mitochondrial dynamic regulation

Besides targeting mitochondrial dysfunction and the Warburg effect, defects such as mitochondrial dynamics and biogenesis are also targets for preclinical treatment. PH therapy can learn from precise medical achievements and reverse the disease process by targeted regulation of metabolic pathways and transcription factors, such as signal transducer and activator of transcription 3 (STAT3), mechanistic target of rapamycin complex (mTORC), Akt, PI3K, FOXO, NFAT, and nuclear factor kappa B (NFκB) (20). Studies have shown that upregulation of HIF-1α/β can activate more than 100 key genes (157) involved in energy metabolism, apoptosis, and so on, which provides a new idea for PH therapy based on tumor/immunotherapy (158). Animal experiments have confirmed that epidermal growth factor receptor (EGFR) and PDGFR inhibitors can significantly improve the hemodynamics and prognosis of PH (159). Molecular mechanism research confirmed that transcription factor KLF5 participated in the occurrence and development of PH by regulating the HIF-1α signaling pathway. Upregulation of G6PD activity promotes the expression of HIF-1α, influencing protein synthesis in PASMCs. Furthermore, elevated expression of STAT3 and HIF-1 is observed in fibroblasts. Recent studies have found that SIRT3 deletion can induce mitochondrial dysfunction and PH occurrence (160), while UCP2 knockout in PASMC promotes vascular remodeling by regulating Ca^2+^ transport (161). BMPR2 signaling pathway participates in PH development by affecting mitochondrial function, and its knock-out can lead to the decrease of ECs mitochondrial membrane potential, ATP synthesis, and the accumulation of mtDNA4977 deletion fragments (162).

Mitochondrial dynamics and biogenesis have also been tried as therapeutic targets in a preclinical environment, although the clinical results are few. The latest research found that activating mitochondrial biogenesis through the NRF-1/HO-1 pathway can significantly improve pulmonary vascular remodeling in experimental animals and reduce RV systolic pressure by about 30% in the chronic hypoxia model (163). At the same time, AMPK pathway activators such as metformin showed double benefits in SU5416-hypoxia rats, not only promoting mitochondrial biogenesis but also improving mitochondrial dynamics (164). Activation of NRF-1, HO-1, and AMPK pathways has shown the potential to improve mitochondrial function and dynamics in experimental animal models, but clinical verification of their therapeutic effect on PH is still needed (163).

### 7.3 PH treatment strategy for mitochondrial oxidative stress

Many preclinical and clinical studies have been carried out to explore the oxidative stress induced by mitochondrial dysfunction. It has been found that in the animal model of PH, a variety of ROS-targeted therapies have shown remarkable efficacy, such as MitoQ compounds that specifically scavenge mitochondrial ROS (165). Although the drug has no obvious improvement effect on chronic hypoxic PH, it can effectively inhibit pulmonary vasoconstriction induced by acute hypoxia, which is a key pathological link in PH progression. In addition, the SOD2 mimetic MitoTEMPO has also been proven to have the regulatory ability to target mitochondrial ROS. It is worth noting that, despite the positive results of preclinical research, including the deer-head-hat rat model, the transformation and application of related therapies in human patients have not yet made a breakthrough. At present, targeted therapy for ROS, such as the SS31 peptide and NRF2 pathway activator, is still in the preclinical research stage (68). In addition, targeting oxidative stress induced by mitochondrial dysfunction has also been tried in preclinical and clinical environments. Drugs such as MitoQ and MitoTEMPO have shown the effect of improving PH phenotype in animal models, but similar results have not been achieved in humans (68). Although there are many pathways related to mitochondrial dysfunction that can be used as therapeutic targets for PH, there is insufficient clinical evidence, which needs further research and verification.

### 7.4 Innovative therapy in the PH field

In the current medical research field, in addition to traditional treatment methods, some emerging therapies have brought new perspectives and possibilities for the treatment of PH (Table 1). On the one hand, mitochondrial transplantation, as a cutting-edge biotechnology, can restore energy metabolism and function by injecting healthy mitochondria into damaged cells, thus improving PH symptoms (166). On the other hand, targeted nano-drugs accurately deliver drugs to the pathological parts of the pulmonary artery by nanotechnology, which improves the curative effect of drugs and reduces systemic side effects. These nano-drugs can carry anti-proliferation, anti-inflammation, or vasodilation drugs, and combine with pulmonary artery ECs or SMC through active or passive targeting mechanisms to realize local drug release and inhibit pulmonary vascular remodeling and inflammatory reaction. Besides, gene editing techniques, such as the CRISPR-Cas9 system, are explored to correct the mutation of genes that cause PH or regulate the expression of related genes, aiming at fundamentally changing the genetic basis of diseases. In particular, it is found that the deletion of the MCJ gene can significantly inhibit the progress of PH, which suggests that the MCJ protein may play an important role in the pathogenesis of PH and provide a potential target for developing new therapeutic drugs targeting the MCJ pathway (167). Besides directly targeting mitochondrial metabolism, modulating the immune-inflammatory response has also emerged as a therapeutic strategy for PH. For example, Rhesus theta defensin-1 (RTD-1), a macrocyclic peptide with immunomodulatory activity, alleviates cytokine storm and acute lung injury by inhibiting the NF-κB and MAPK pathways. Preclinical studies have confirmed its favorable pharmacokinetic profile and safety, providing a rationale for its potential use in treating PH-associated pulmonary inflammation (168).

**TABLE 1:** Evidence grading of mitochondrial targeted therapy in pulmonary hypertension.

Target/pathway	Intervention measure	Research stage	Models/crowds	Main findings	Safety/limitations
**Glycolysis/OXPHOS imbalance**					
PDK → PDH	DCA	Preclinical (*In vitro*/animal)	Mouse models	Reversing Warburg effect, improving pulmonary vascular remodeling	Effective preclinically
		Phase II clinical trial (Human)	PAH patients	The potential to improve hemodynamics	Requires larger trials for validation
PFKFB3/ENO 1	Inhibitor	Preclinical (Animal)	Animal models	Inhibition of PAH development	Data limited to animal models
FAO	Ranolazine	Preclinical (Animal)	Mouse models	Showed significant protective effect	Limited clinical data
		Phase II clinical trial (Human)	PAH patients	Did not reduce mPAP	Failed to meet the primary hemodynamic endpoint
FAO	Trimetazidine	Preclinical (animal)	Rat models	Inhibited PASMC proliferation	Effective preclinically
		Under clinical investigation (human)	Experimental phase	May act by preserving mitochondrial function	Clinical evidence is still accumulating
Calcium signaling/NFAT	Cyclosporine A	Preclinical (animal)	Rat model	Reduced mPAP, improved vascular remodeling index	Significant individual variability, risk of renal impairment
		Phase II clinical trial (Human)	PAH patients	Efficacy varied among individuals	
**Mitochondrial dynamics and biogenesis**					
NRF-1/HO-1 pathway	Pathway activators	Preclinical (animal)	Animal model	Improved pulmonary vascular remodeling, reduced RVSP	Data limited to animal models
AMPK pathway	Metformin	Preclinical (animal)	Rat model	Promoted mitochondrial biogenesis, improved mitochondrial dynamics	Data limited to animal models
**Mitochondrial oxidative stress**					
mROS	MitoQ	Preclinical (animal)	Animal models	Effectively inhibited acute HPV	No significant improvement in chronic hypoxic PH
	MitoTEMPO	Preclinical (animal)	Animal models	Demonstrated ability to modulate mROS	Data limited to animal models
mROS/NRF2 pathway	SS-31 Peptide/NRF2 activators	Preclinical (research phase)	Preclinical models	Still in the preclinical research stage	Lacks clinical data

AMPK, AMP-activated Protein Kinase; DCA, Dichloroacetate; ENO1, Enolase 1; FAO, Fatty Acid Oxidation; HO-1, Heme Oxygenase-1; HPV, Hypoxia-Induced Pulmonary Vasoconstriction; mROS, Mitochondrial Reactive Oxygen Species; NRF-1, Nuclear Respiratory Factor 1; NRF2, Nuclear Factor Erythroid 2–Related Factor 2; NFAT, Nuclear Factor of Activated T-cells; OXPHOS, Oxidative Phosphorylation; PAH, Pulmonary Arterial Hypertension; PASMC, Pulmonary Artery Smooth Muscle Cell; PDK, Pyruvate Dehydrogenase Kinase; PDH, Pyruvate Dehydrogenase; PFKFB3, 6-Phosphofructo-2-Kinase/Fructose-2,6-Bisphosphatase 3; RVSP, Right Ventricular Systolic Pressure.

### 7.5 Controversies and challenges in mitochondrial-targeted therapies

Despite the promising preclinical data, the therapeutic efficacy of several mitochondrial-targeted drugs in PH remains a subject of ongoing debate, highlighting the challenges in translating bench findings to bedside application. For instance, while DCA demonstrates efficacy in animal models, its long-term benefits and potential neurotoxicity in humans warrant careful consideration (149). Similarly, the mixed results from clinical trials of FAO inhibitors like ranolazine—which improved functional parameters but failed to significantly reduce mPAP—underscore the complexity of metabolic reprogramming in PH and the potential disconnect between hemodynamic and clinical endpoints (151). Furthermore, although antioxidants such as MitoQ showed efficacy in acute settings, their inability to ameliorate chronic hypoxic PH phenotypes suggests that the timing, context, and specific molecular sources of oxidative stress are critical determinants of treatment success (68, 165). These discrepancies may arise from species-specific differences, disease heterogeneity in human PH, or compensatory mechanisms that bypass single-target inhibition. Therefore, future efforts should prioritize patient stratification, combination therapies targeting parallel pathways, and the development of more precise methods to monitor mitochondrial function *in vivo*.

## 8 Conclusion

More evidence shows that mitochondrial dysfunction plays a significant role in PH. Interventions targeting the mitochondrial quality control system, such as adjusting mitochondrial dynamics, improving OXPHOS efficiency, or using mitochondrial-targeted antioxidants, may constitute a new direction for treating PH. To effectively diagnose and treat PH, it is particularly important to further investigate the regulatory mechanisms of mitochondrial dysfunction. Based on an understanding of these pathways, future research will likely adopt more systematic and targeted strategies to address the complex and heterogeneous nature of this disease.

It is further noted that despite significant progress in existing research findings, their integration into clinical practice remains challenging. On one hand, most of these findings are still within a pre-clinical stage, lacking enough clinical trial data for direct implementation. On the other hand, current treatment guidelines often lack precise target definitions. Therefore, future research should delve deeper into mitochondrial mechanism studies concerning PH diseases and evaluate the optimal application opportunity of mitochondrial-targeted drugs with high efficacy. In addition, the long-term safety and effectiveness of such drugs also need to be systematically evaluated and analyzed.

## References

[1] Colon Hidalgo D Elajaili H Suliman H George M Delaney C Nozik E. Metabolism, mitochondrial dysfunction, and redox homeostasis in pulmonary hypertension. *Antioxidants.* (2022) 11:428. 10.3390/antiox11020428 35204311 PMC8869288

[2] Humbert M Kovacs G Hoeper M Badagliacca R Berger R Brida M 2022 ESC/ERS Guidelines for the diagnosis and treatment of pulmonary hypertension. *Eur Heart J.* (2022) 43:3618–731. 10.1093/eurheartj/ehac237 36017548

[3] Kierans S Taylor C. Regulation of glycolysis by the hypoxia-inducible factor (HIF): implications for cellular physiology. *J Physiol.* (2021) 599:23–37. 10.1113/jp280572 33006160

[4] Zhang W Liu B Wang Y Zhang H He L Wang P Mitochondrial dysfunction in pulmonary arterial hypertension. *Front Physiol.* (2022) 13:1079989. 10.3389/fphys.2022.1079989 36589421 PMC9795033

[5] Galiè N Channick R Frantz R Grünig E Jing Z Moiseeva O Risk stratification and medical therapy of pulmonary arterial hypertension. *Eur Respir J.* (2019) 53:1801889. 10.1183/13993003.01889-2018 30545971 PMC6351343

[6] Simonneau G Gatzoulis M Adatia I Celermajer D Denton C Ghofrani A Updated clinical classification of pulmonary hypertension. *J Am Coll Cardiol.* (2013) 62:D34–41. 10.1016/j.jacc.2013.10.029 24355639

[7] Torres-Arrese M Barberá-Rausell P Li-Zhu J Salas-Dueñas R Real-Martín A Mata-Martínez A The cardiac pulsed wave doppler pattern of the common femoral vein in diagnosing the likelihood of severe pulmonary hypertension: results from a prospective multicentric study. *J Clin Med.* (2024) 13:3860. 10.3390/jcm13133860 38999425 PMC11242826

[8] Douschan P Kaemmerer-Suleiman A Egenlauf B Seyfarth H Zeder K Mayer L Definition und Klassifikation der pulmonalen Hypertonie [Definition and classification of pulmonary hypertension]. *Pneumologie.* (2025) 79:723–31. German. 10.1055/a-2625-4769 41052526

[9] Hoeper M. Definition, classification, and epidemiology of pulmonary arterial hypertension. *Semin Respir Crit Care Med.* (2009) 30:369–75. 10.1055/s-0029-1233306 19634076

[10] Chan S Loscalzo J. Pathogenic mechanisms of pulmonary arterial hypertension. *J Mol Cell Cardiol.* (2008) 44:14–30. 10.1016/j.yjmcc.2007.09.006 17950310 PMC2234575

[11] Shimoda L Laurie S. Vascular remodeling in pulmonary hypertension. *J Mol Med.* (2013) 91:297–309. 10.1007/s00109-013-0998-0 23334338 PMC3584237

[12] Hoeper M Humbert M Souza R Idrees M Kawut S Sliwa-Hahnle K A global view of pulmonary hypertension. *Lancet Respir Med.* (2016) 4:306–22. 10.1016/s2213-2600(15)00543-3 26975810

[13] Hatton N Ryan J. Sex differences in response to pulmonary arterial hypertension therapy: is what’s good for the goose, good for the gander? *Chest.* (2014) 145:1184–6. 10.1378/chest.13-3061 24889427

[14] Campo A Mathai S Le Pavec J Zaiman A Hummers L Boyce D Outcomes of hospitalisation for right heart failure in pulmonary arterial hypertension. *Eur Respir J.* (2011) 38:359–67. 10.1183/09031936.00148310 21310884

[15] Mocumbi A Humbert M Saxena A Jing Z Sliwa K Thienemann F Pulmonary hypertension. *Nat Rev Dis Primers.* (2024) 10:1. 10.1038/s41572-023-00486-7 38177157

[16] Hautefort A Mendes-Ferreira P Sabourin J Manaud G Bertero T Rucker-Martin C Bmpr2 mutant rats develop pulmonary and cardiac characteristics of pulmonary arterial hypertension. *Circulation.* (2019) 139:932–48. 10.1161/circulationaha.118.033744 30586714

[17] Dumas S Bru-Mercier G Courboulin A Quatredeniers M Rücker-Martin C Antigny F NMDA-type glutamate receptor activation promotes vascular remodeling and pulmonary arterial hypertension. *Circulation.* (2018) 137:2371–89. 10.1161/circulationaha.117.029930 29444988

[18] Lambert M Capuano V Boet A Tesson L Bertero T Nakhleh M Characterization of Kcnk3-mutated rat, a novel model of pulmonary hypertension. *Circ Res.* (2019) 125:678–95. 10.1161/circresaha.119.314793 31347976

[19] Siques P Pena E Brito J El Alam S. Oxidative stress, kinase activation, and inflammatory pathways involved in effects on smooth muscle cells during pulmonary artery hypertension under hypobaric hypoxia exposure. *Front Physiol.* (2021) 12:690341. 10.3389/fphys.2021.690341 34434114 PMC8381601

[20] Harvey L Chan S. Emerging metabolic therapies in pulmonary arterial hypertension. *J Clin Med.* (2017) 6:43. 10.3390/jcm6040043 28375184 PMC5406775

[21] Cloonan S Choi A. Mitochondria in lung disease. *J Clin Invest.* (2016) 126:809–20. 10.1172/jci81113 26928034 PMC4767339

[22] Veyssier-Belot C Cacoub P. Role of endothelial and smooth muscle cells in the physiopathology and treatment management of pulmonary hypertension. *Cardiovasc Res.* (1999) 44:274–82. 10.1016/s0008-6363(99)00230-8 10690304

[23] Guignabert C Tu L Girerd B Ricard N Huertas A Montani D New molecular targets of pulmonary vascular remodeling in pulmonary arterial hypertension: importance of endothelial communication. *Chest.* (2015) 147:529–37. 10.1378/chest.14-0862 25644906

[24] Dunham-Snary K Wu D Sykes E Thakrar A Parlow L Mewburn J Hypoxic pulmonary vasoconstriction: from molecular mechanisms to medicine. *Chest.* (2017) 151:181–92. 10.1016/j.chest.2016.09.001 27645688 PMC5310129

[25] Ranchoux B Harvey L Ayon R Babicheva A Bonnet S Chan S Endothelial dysfunction in pulmonary arterial hypertension: an evolving landscape (2017 Grover Conference Series). *Pulm Circ.* (2018) 8:2045893217752912. 10.1177/2045893217752912 29283043 PMC5798691

[26] Humbert M Guignabert C Bonnet S Dorfmüller P Klinger J Nicolls M Pathology and pathobiology of pulmonary hypertension: state of the art and research perspectives. *Eur Respir J.* (2019) 53:1801887. 10.1183/13993003.01887-2018 30545970 PMC6351340

[27] Wang R Yuan T Wang J Chen Y Zhao J Li M Immunity and inflammation in pulmonary arterial hypertension: from pathophysiology mechanisms to treatment perspective. *Pharmacol Res.* (2022) 180:106238. 10.1016/j.phrs.2022.106238 35504356

[28] Kherbeck N Tamby M Bussone G Dib H Perros F Humbert M The role of inflammation and autoimmunity in the pathophysiology of pulmonary arterial hypertension. *Clin Rev Allergy Immunol.* (2013) 44:31–8. 10.1007/s12016-011-8265-z 21394427

[29] Balk A Dingemans K Wagenvoort C. The ultrastructure of the various forms of pulmonary arterial intimal fibrosis. *Virchows Arch A Pathol Anat Histol.* (1979) 382:139–50. 10.1007/bf01102870 157603

[30] Oka M Homma N Taraseviciene-Stewart L Morris K Kraskauskas D Burns N Rho kinase-mediated vasoconstriction is important in severe occlusive pulmonary arterial hypertension in rats. *Circ Res.* (2007) 100:923–9. 10.1161/01.Res.0000261658.12024.18 17332430

[31] Malenfant S Neyron A Paulin R Potus F Meloche J Provencher S Signal transduction in the development of pulmonary arterial hypertension. *Pulm Circ.* (2013) 3:278–93. 10.4103/2045-8932.114752 24015329 PMC3757823

[32] Ruopp N Cockrill B. Diagnosis and treatment of pulmonary arterial hypertension: a review. *JAMA.* (2022) 327:1379–91. 10.1001/jama.2022.4402 35412560

[33] Howard L. Prognostic factors in pulmonary arterial hypertension: assessing the course of the disease. *Eur Respir Rev.* (2011) 20:236–42. 10.1183/09059180.00006711 22130816 PMC9487744

[34] Humbert M Sitbon O Chaouat A Bertocchi M Habib G Gressin V Survival in patients with idiopathic, familial, and anorexigen-associated pulmonary arterial hypertension in the modern management era. *Circulation.* (2010) 122:156–63. 10.1161/circulationaha.109.911818 20585011

[35] Bogaard H Natarajan R Henderson S Long C Kraskauskas D Smithson L Chronic pulmonary artery pressure elevation is insufficient to explain right heart failure. *Circulation.* (2009) 120:1951–60. 10.1161/circulationaha.109.883843 19884466

[36] Decker I Ghosh S Comhair S Farha S Tang W Park M High levels of zinc-protoporphyrin identify iron metabolic abnormalities in pulmonary arterial hypertension. *Clin Transl Sci.* (2011) 4:253–8. 10.1111/j.1752-8062.2011.00301.x 21884511 PMC3575639

[37] Michelakis ED Thébaud B Weir E Archer S. Hypoxic pulmonary vasoconstriction: redox regulation of O2-sensitive K+ channels by a mitochondrial O2-sensor in resistance artery smooth muscle cells. *J Mol Cell Cardiol.* (2004) 37:1119–36. 10.1016/j.yjmcc.2004.09.007 15572043

[38] Suliman H Nozik-Grayck E. Mitochondrial dysfunction: metabolic drivers of pulmonary hypertension. *Antioxid Redox Signal.* (2019) 31:843–57. 10.1089/ars.2018.7705 30604624 PMC6751393

[39] Kwong J Molkentin J. Physiological and pathological roles of the mitochondrial permeability transition pore in the heart. *Cell Metab.* (2015) 21:206–14. 10.1016/j.cmet.2014.12.001 25651175 PMC4616258

[40] Dromparis P Michelakis ED. Mitochondria in vascular health and disease. *Annu Rev Physiol.* (2013) 75:95–126. 10.1146/annurev-physiol-030212-183804 23157555

[41] Hackenbrock C. Ultrastructural bases for metabolically linked mechanical activity in mitochondria. II. Electron transport-linked ultrastructural transformations in mitochondria. *J Cell Biol.* (1968) 37:345–69. 10.1083/jcb.37.2.345 5656397 PMC2107416

[42] Chinnery P Elliott H Hudson G Samuels D Relton C. Epigenetics, epidemiology and mitochondrial DNA diseases. *Int J Epidemiol.* (2012) 41:177–87. 10.1093/ije/dyr232 22287136 PMC3304530

[43] Zhang Q Raoof M Chen Y Sumi Y Sursal T Junger W Circulating mitochondrial DAMPs cause inflammatory responses to injury. *Nature.* (2010) 464:104–7. 10.1038/nature08780 20203610 PMC2843437

[44] Borcherding N Brestoff J. The power and potential of mitochondria transfer. *Nature.* (2023) 623:283–91. 10.1038/s41586-023-06537-z 37938702 PMC11590279

[45] Sansone P Savini C Kurelac I Chang Q Amato L Strillacci A Packaging and transfer of mitochondrial DNA via exosomes regulate escape from dormancy in hormonal therapy-resistant breast cancer. *Proc Natl Acad Sci U S A.* (2017) 114:E9066–75. 10.1073/pnas.1704862114 29073103 PMC5664494

[46] Bhatti J Bhatti G Reddy P. Mitochondrial dysfunction and oxidative stress in metabolic disorders - A step towards mitochondria based therapeutic strategies. *Biochim Biophys Acta Mol Basis Dis.* (2017) 1863:1066–77. 10.1016/j.bbadis.2016.11.010 27836629 PMC5423868

[47] Griffiths E. Mitochondria–potential role in cell life and death. *Cardiovasc Res.* (2000) 46:24–7. 10.1016/s0008-6363(00)00020-1 10727650

[48] Culley M Chan S. Mitochondrial metabolism in pulmonary hypertension: beyond mountains there are mountains. *J Clin Invest.* (2018) 128:3704–15. 10.1172/jci120847 30080181 PMC6118596

[49] Ryanto G Suraya R Nagano T. Mitochondrial dysfunction in pulmonary hypertension. *Antioxidants.* (2023) 12:372. 10.3390/antiox12020372 36829931 PMC9952650

[50] Wilkins M. Pulmonary hypertension: the science behind the disease spectrum. *Eur Respir Rev.* (2012) 21:19–26. 10.1183/09059180.00008411 22379170 PMC9487470

[51] Nunnari J Suomalainen A. Mitochondria: in sickness and in health. *Cell.* (2012) 148:1145–59. 10.1016/j.cell.2012.02.035 22424226 PMC5381524

[52] Zong Y Li H Liao P Chen L Pan Y Zheng Y Mitochondrial dysfunction: mechanisms and advances in therapy. *Signal Transduct Target Ther.* (2024) 9:124. 10.1038/s41392-024-01839-8 38744846 PMC11094169

[53] Ryan J Archer S. Emerging concepts in the molecular basis of pulmonary arterial hypertension: part I: metabolic plasticity and mitochondrial dynamics in the pulmonary circulation and right ventricle in pulmonary arterial hypertension. *Circulation.* (2015) 131:1691–702. 10.1161/circulationaha.114.006979 25964279 PMC4429908

[54] Tuder R. Pulmonary vascular remodeling in pulmonary hypertension. *Cell Tissue Res.* (2017) 367:643–9. 10.1007/s00441-016-2539-y 28025704 PMC5408737

[55] Riou M Enache I Sauer F Charles A Geny B. Targeting mitochondrial metabolic dysfunction in pulmonary hypertension: toward new therapeutic approaches? *Int J Mol Sci.* (2023) 24:9572. 10.3390/ijms24119572 37298522 PMC10253387

[56] Pieczenik S Neustadt J. Mitochondrial dysfunction and molecular pathways of disease. *Exp Mol Pathol.* (2007) 83:84–92. 10.1016/j.yexmp.2006.09.008 17239370

[57] Rafikov R Sun X Rafikova O Louise Meadows M Desai A Khalpey Z Complex I dysfunction underlies the glycolytic switch in pulmonary hypertensive smooth muscle cells. *Redox Biol.* (2015) 6:278–86. 10.1016/j.redox.2015.07.016 26298201 PMC4556771

[58] Breault N Wu D Dasgupta A Chen K Archer S. Acquired disorders of mitochondrial metabolism and dynamics in pulmonary arterial hypertension. *Front Cell Dev Biol.* (2023) 11:1105565. 10.3389/fcell.2023.1105565 36819102 PMC9933518

[59] Ma C Wang X He S Zhang L Bai J Qu L Ubiquitinated AIF is a major mediator of hypoxia-induced mitochondrial dysfunction and pulmonary artery smooth muscle cell proliferation. *Cell Biosci.* (2022) 12:9. 10.1186/s13578-022-00744-3 35090552 PMC8796423

[60] Adesina S Kang B Bijli K Ma J Cheng J Murphy T Targeting mitochondrial reactive oxygen species to modulate hypoxia-induced pulmonary hypertension. *Free Radic Biol Med.* (2015) 87:36–47. 10.1016/j.freeradbiomed.2015.05.042 26073127 PMC4615392

[61] Bretón-Romero R Lamas S. Hydrogen peroxide signaling in vascular endothelial cells. *Redox Biol.* (2014) 2:529–34. 10.1016/j.redox.2014.02.005 24634835 PMC3953958

[62] Liang S Yegambaram M Wang T Wang J Black S Tang H. Mitochondrial metabolism, redox, and calcium homeostasis in pulmonary arterial hypertension. *Biomedicines.* (2022) 10:341. 10.3390/biomedicines10020341 35203550 PMC8961787

[63] Wallace D. Colloquium paper: bioenergetics, the origins of complexity, and the ascent of man. *Proc Natl Acad Sci U S A.* (2010) 107:8947–53. 10.1073/pnas.0914635107 20445102 PMC3024017

[64] Brand M Nicholls D. Assessing mitochondrial dysfunction in cells. *Biochem J.* (2011) 435:297–312. 10.1042/bj20110162 21726199 PMC3076726

[65] Rossignol R Faustin B Rocher C Malgat M Mazat J Letellier T. Mitochondrial threshold effects. *Biochem J.* (2003) 370:751–62. 10.1042/bj20021594 12467494 PMC1223225

[66] West A Shadel G. Mitochondrial DNA in innate immune responses and inflammatory pathology. *Nat Rev Immunol.* (2017) 17:363–75. 10.1038/nri.2017.21 28393922 PMC7289178

[67] Potoka K Gladwin M. Vasculopathy and pulmonary hypertension in sickle cell disease. *Am J Physiol Lung Cell Mol Physiol.* (2015) 308:L314–24. 10.1152/ajplung.00252.2014 25398989 PMC4329471

[68] Marshall J Bazan I Zhang Y Fares W Lee P. Mitochondrial dysfunction and pulmonary hypertension: cause, effect, or both. *Am J Physiol Lung Cell Mol Physiol.* (2018) 314:L782–96. 10.1152/ajplung.00331.2017 29345195 PMC6008129

[69] Schumacker P Gillespie M Nakahira K Choi A Crouser ED Piantadosi C Mitochondria in lung biology and pathology: more than just a powerhouse. *Am J Physiol Lung Cell Mol Physiol.* (2014) 306:L962–74. 10.1152/ajplung.00073.2014 24748601 PMC4042189

[70] Ruchko M Gorodnya O LeDoux S Alexeyev M Al-Mehdi A Gillespie M. Mitochondrial DNA damage triggers mitochondrial dysfunction and apoptosis in oxidant-challenged lung endothelial cells. *Am J Physiol Lung Cell Mol Physiol.* (2005) 288:L530–5. 10.1152/ajplung.00255.2004 15563690

[71] Hashizume M Mouner M Chouteau J Gorodnya O Ruchko M Potter B Mitochondrial-targeted DNA repair enzyme 8-oxoguanine DNA glycosylase 1 protects against ventilator-induced lung injury in intact mice. *Am J Physiol Lung Cell Mol Physiol.* (2013) 304:L287–97. 10.1152/ajplung.00071.2012 23241530 PMC3567361

[72] Chouteau J Obiako B Gorodnya O Pastukh V Ruchko M Wright A Mitochondrial DNA integrity may be a determinant of endothelial barrier properties in oxidant-challenged rat lungs. *Am J Physiol Lung Cell Mol Physiol.* (2011) 301:L892–8. 10.1152/ajplung.00210.2011 21890512 PMC3233824

[73] Li J Yang F Wei F Ren X. The role of toll-like receptor 4 in tumor microenvironment. *Oncotarget.* (2017) 8:66656–67. 10.18632/oncotarget.19105 29029545 PMC5630445

[74] Kuck J Obiako B Gorodnya O Pastukh V Kua J Simmons J Mitochondrial DNA damage-associated molecular patterns mediate a feed-forward cycle of bacteria-induced vascular injury in perfused rat lungs. *Am J Physiol Lung Cell Mol Physiol.* (2015) 308:L1078–85. 10.1152/ajplung.00015.2015 25795724 PMC4437009

[75] Yin J You S Liu H Chen L Zhang C Hu H Role of P2X(7)R in the development and progression of pulmonary hypertension. *Respir Res.* (2017) 18:127. 10.1186/s12931-017-0603-0 28646872 PMC5483271

[76] Xu S Xu X Zhang J Ying K Shao Y Zhang R. Pulmonary hypertension as a manifestation of mitochondrial disease: a case report and review of the literature. *Medicine.* (2017) 96:e8716. 10.1097/md.0000000000008716 29145311 PMC5704856

[77] Dai J Zhou Q Chen J Rexius-Hall M Rehman J Zhou G. Alpha-enolase regulates the malignant phenotype of pulmonary artery smooth muscle cells via the AMPK-Akt pathway. *Nat Commun.* (2018) 9:3850. 10.1038/s41467-018-06376-x 30242159 PMC6155017

[78] Xu W Koeck T Lara A Neumann D DiFilippo F Koo M Alterations of cellular bioenergetics in pulmonary artery endothelial cells. *Proc Natl Acad Sci U S A.* (2007) 104:1342–7. 10.1073/pnas.0605080104 17227868 PMC1783136

[79] Sutendra G Dromparis P Wright P Bonnet S Haromy A Hao Z The role of Nogo and the mitochondria-endoplasmic reticulum unit in pulmonary hypertension. *Sci Transl Med.* (2011) 3:88ra55. 10.1126/scitranslmed.3002194 21697531 PMC3744110

[80] Teichert-Kuliszewska K Kutryk M Kuliszewski M Karoubi G Courtman D Zucco L Bone morphogenetic protein receptor-2 signaling promotes pulmonary arterial endothelial cell survival: implications for loss-of-function mutations in the pathogenesis of pulmonary hypertension. *Circ Res.* (2006) 98:209–17. 10.1161/01.RES.0000200180.01710.e6 16357305

[81] Dromparis P Sutendra G Michelakis ED. The role of mitochondria in pulmonary vascular remodeling. *J Mol Med.* (2010) 88:1003–10. 10.1007/s00109-010-0670-x 20734021

[82] Roberts R Laskin D Smith C Robertson F Allen E Doorn J Nitrative and oxidative stress in toxicology and disease. *Toxicol Sci.* (2009) 112:4–16. 10.1093/toxsci/kfp179 19656995 PMC2769059

[83] Zorova L Popkov V Plotnikov E Silachev D Pevzner I Jankauskas S Mitochondrial membrane potential. *Anal Biochem.* (2018) 552:50–9. 10.1016/j.ab.2017.07.009 28711444 PMC5792320

[84] Owen O Kalhan S Hanson R. The key role of anaplerosis and cataplerosis for citric acid cycle function. *J Biol Chem.* (2002) 277:30409–12. 10.1074/jbc.R200006200 12087111

[85] Li D Shao N Moonen J Zhao Z Shi M Otsuki S ALDH1A3 coordinates metabolism with gene regulation in pulmonary arterial hypertension. *Circulation.* (2021) 143:2074–90. 10.1161/circulationaha.120.048845 33764154 PMC8289565

[86] Semenza G. Hypoxia-inducible factors in physiology and medicine. *Cell.* (2012) 148:399–408. 10.1016/j.cell.2012.01.021 22304911 PMC3437543

[87] Arts R Novakovic B Ter Horst R Carvalho A Bekkering S Lachmandas E Glutaminolysis and fumarate accumulation integrate immunometabolic and epigenetic programs in trained immunity. *Cell Metab.* (2016) 24:807–19. 10.1016/j.cmet.2016.10.008 27866838 PMC5742541

[88] Ballinger S. Mitochondrial dysfunction in cardiovascular disease. *Free Radic Biol Med.* (2005) 38:1278–95. 10.1016/j.freeradbiomed.2005.02.014 15855047

[89] Cadenas S. ROS and redox signaling in myocardial ischemia-reperfusion injury and cardioprotection. *Free Radic Biol Med.* (2018) 117:76–89. 10.1016/j.freeradbiomed.2018.01.024 29373843

[90] Ryan J Dasgupta A Huston J Chen K Archer S. Mitochondrial dynamics in pulmonary arterial hypertension. *J Mol Med.* (2015) 93:229–42. 10.1007/s00109-015-1263-5 25672499 PMC4339102

[91] Picca A Lezza A. Regulation of mitochondrial biogenesis through TFAM-mitochondrial DNA interactions: useful insights from aging and calorie restriction studies. *Mitochondrion.* (2015) 25:67–75. 10.1016/j.mito.2015.10.001 26437364

[92] Popov L. Mitochondrial biogenesis: an update. *J Cell Mol Med.* (2020) 24:4892–9. 10.1111/jcmm.15194 32279443 PMC7205802

[93] Wang S Long H Hou L Feng B Ma Z Wu Y The mitophagy pathway and its implications in human diseases. *Signal Transduct Target Ther.* (2023) 8:304. 10.1038/s41392-023-01503-7 37582956 PMC10427715

[94] Jornayvaz F Shulman G. Regulation of mitochondrial biogenesis. *Essays Biochem.* (2010) 47:69–84. 10.1042/bse0470069 20533901 PMC3883043

[95] Cilleros-Holgado P Gómez-Fernández D Piñero-Pérez R Romero-Domínguez J Reche-López D López-Cabrera A Mitochondrial quality control via mitochondrial unfolded protein response (mtUPR) in ageing and neurodegenerative diseases. *Biomolecules.* (2023) 13:1789. 10.3390/biom13121789 38136659 PMC10741690

[96] Yoo S Jung YK. A Molecular approach to mitophagy and mitochondrial dynamics. *Mol Cells.* (2018) 41:18–26. 10.14348/molcells.2018.2277 29370689 PMC5792708

[97] Klaips C Jayaraj G Hartl F. Pathways of cellular proteostasis in aging and disease. *J Cell Biol.* (2018) 217:51–63. 10.1083/jcb.201709072 29127110 PMC5748993

[98] Wang S Kaufman R. The impact of the unfolded protein response on human disease. *J Cell Biol.* (2012) 197:857–67. 10.1083/jcb.201110131 22733998 PMC3384412

[99] Ren L Chen X Chen X Li J Cheng B Xia J. Mitochondrial dynamics: fission and fusion in fate determination of mesenchymal stem cells. *Front Cell Dev Biol.* (2020) 8:580070. 10.3389/fcell.2020.580070 33178694 PMC7593605

[100] Venkatesh S Li M Saito T Tong M Rashed E Mareedu S Mitochondrial LonP1 protects cardiomyocytes from ischemia/reperfusion injury in vivo. *J Mol Cell Cardiol.* (2019) 128:38–50. 10.1016/j.yjmcc.2018.12.017 30625302

[101] Youle R van der Bliek A. Mitochondrial fission, fusion, and stress. *Science.* (2012) 337:1062–5. 10.1126/science.1219855 22936770 PMC4762028

[102] Liesa M Palacín M Zorzano A. Mitochondrial dynamics in mammalian health and disease. *Physiol Rev.* (2009) 89:799–845. 10.1152/physrev.00030.2008 19584314

[103] Tilokani L Nagashima S Paupe V Prudent J. Mitochondrial dynamics: overview of molecular mechanisms. *Essays Biochem.* (2018) 62:341–60. 10.1042/ebc20170104 30030364 PMC6056715

[104] Kumari S Singh K Khadia M Kumar R Bansal V Mishra A. Exploring the influence of metabolic changes in fibrotic lung diseases. *Pulm Circ.* (2025) 15:e70163. 10.1002/pul2.70163 40989087 PMC12452044

[105] Santos E Khatoon S Di Mise A Zheng Y Wang Y. Mitochondrial dynamics in pulmonary hypertension. *Biomedicines.* (2023) 12:53. 10.3390/biomedicines12010053 38255160 PMC10813473

[106] Song Z Ghochani M McCaffery J Frey T Chan D. Mitofusins and OPA1 mediate sequential steps in mitochondrial membrane fusion. *Mol Biol Cell.* (2009) 20:3525–32. 10.1091/mbc.e09-03-0252 19477917 PMC2719570

[107] Chen H McCaffery J Chan D. Mitochondrial fusion protects against neurodegeneration in the cerebellum. *Cell.* (2007) 130:548–62. 10.1016/j.cell.2007.06.026 17693261

[108] Bergeron R Ren J Cadman K Moore I Perret P Pypaert M Chronic activation of AMP kinase results in NRF-1 activation and mitochondrial biogenesis. *Am J Physiol Endocrinol Metab.* (2001) 281:E1340–6. 10.1152/ajpendo.2001.281.6.E1340 11701451

[109] Zong H Ren J Young L Pypaert M Mu J Birnbaum M AMP kinase is required for mitochondrial biogenesis in skeletal muscle in response to chronic energy deprivation. *Proc Natl Acad Sci U S A.* (2002) 99:15983–7. 10.1073/pnas.252625599 12444247 PMC138551

[110] Otera H Wang C Cleland M Setoguchi K Yokota S Youle R Mff is an essential factor for mitochondrial recruitment of Drp1 during mitochondrial fission in mammalian cells. *J Cell Biol.* (2010) 191:1141–58. 10.1083/jcb.201007152 21149567 PMC3002033

[111] Palmer C Osellame L Laine D Koutsopoulos O Frazier A Ryan M. MiD49 and MiD51, new components of the mitochondrial fission machinery. *EMBO Rep.* (2011) 12:565–73. 10.1038/embor.2011.54 21508961 PMC3128275

[112] Rehman J Zhang H Toth P Zhang Y Marsboom G Hong Z Inhibition of mitochondrial fission prevents cell cycle progression in lung cancer. *Faseb J.* (2012) 26:2175–86. 10.1096/fj.11-196543 22321727 PMC3336787

[113] Waxman A Restrepo-Jaramillo R Thenappan T Ravichandran A Engel P Bajwa A Inhaled Treprostinil in pulmonary hypertension due to interstitial lung disease. *N Engl J Med.* (2021) 384:325–34. 10.1056/NEJMoa2008470 33440084

[114] Abu-Hanna J Anastasakis E Patel J Eddama M Denton C Taanman J Prostacyclin mimetics inhibit DRP1-mediated pro-proliferative mitochondrial fragmentation in pulmonary arterial hypertension. *Vascul Pharmacol.* (2023) 151:107194. 10.1016/j.vph.2023.107194 37442283

[115] Feng W Wang J Yan X Zhang Q Chai L Wang Q ERK/Drp1-dependent mitochondrial fission contributes to HMGB1-induced autophagy in pulmonary arterial hypertension. *Cell Prolif.* (2021) 54:e13048. 10.1111/cpr.13048 33948998 PMC8168414

[116] Ryan J Marsboom G Fang Y Toth P Morrow E Luo N PGC1α-mediated mitofusin-2 deficiency in female rats and humans with pulmonary arterial hypertension. *Am J Respir Crit Care Med.* (2013) 187:865–78. 10.1164/rccm.201209-1687OC 23449689 PMC3707374

[117] Stewart D Levy R Cernacek P Langleben D. Increased plasma endothelin-1 in pulmonary hypertension: marker or mediator of disease? *Ann Intern Med.* (1991) 114:464–9. 10.7326/0003-4819-114-6-464 1994793

[118] Chung K Hsu C Fan L Huang Z Bhatia D Chen Y Mitofusins regulate lipid metabolism to mediate the development of lung fibrosis. *Nat Commun.* (2019) 10:3390. 10.1038/s41467-019-11327-1 31358769 PMC6662701

[119] Ding Q Qi Y Tsang S. Mitochondrial biogenesis, mitochondrial dynamics, and mitophagy in the maturation of cardiomyocytes. *Cells.* (2021) 10:2463. 10.3390/cells10092463 34572112 PMC8466139

[120] Sithamparanathan S Rocha M Parikh J Rygiel K Falkous G Grady J Skeletal muscle mitochondrial oxidative phosphorylation function in idiopathic pulmonary arterial hypertension: in vivo and in vitro study. *Pulm Circ.* (2018) 8:2045894018768290. 10.1177/2045894018768290 29799315 PMC5971390

[121] Piantadosi C Suliman H. Redox regulation of mitochondrial biogenesis. *Free Radic Biol Med.* (2012) 53:2043–53. 10.1016/j.freeradbiomed.2012.09.014 23000245 PMC3604744

[122] Gureev A Shaforostova E Popov V. Regulation of mitochondrial biogenesis as a way for active longevity: interaction between the Nrf2 and PGC-1α signaling pathways. *Front Genet.* (2019) 10:435. 10.3389/fgene.2019.00435 31139208 PMC6527603

[123] Fernandez-Marcos P Auwerx J. Regulation of PGC-1α, a nodal regulator of mitochondrial biogenesis. *Am J Clin Nutr.* (2011) 93:884s–90. 10.3945/ajcn.110.001917 21289221 PMC3057551

[124] Ventura-Clapier R Garnier A Veksler V. Transcriptional control of mitochondrial biogenesis: the central role of PGC-1alpha. *Cardiovasc Res.* (2008) 79:208–17. 10.1093/cvr/cvn098 18430751

[125] Suliman H Piantadosi C. Mitochondrial quality control as a therapeutic target. *Pharmacol Rev.* (2016) 68:20–48. 10.1124/pr.115.011502 26589414 PMC11060432

[126] Sisniega C Zayas N Pulido T. Advances in medical therapy for pulmonary arterial hypertension. *Curr Opin Cardiol.* (2019) 34:98–103. 10.1097/hco.0000000000000583 30394906

[127] Chen Y Yuan T Zhang H Yan Y Wang D Fang L Activation of Nrf2 attenuates pulmonary vascular remodeling via inhibiting endothelial-to-mesenchymal transition: an insight from a plant polyphenol. *Int J Biol Sci.* (2017) 13:1067–81. 10.7150/ijbs.20316 28924387 PMC5599911

[128] Vega R Horton J Kelly D. Maintaining ancient organelles: mitochondrial biogenesis and maturation. *Circ Res.* (2015) 116:1820–34. 10.1161/circresaha.116.305420 25999422 PMC4443496

[129] Waypa G Osborne S Marks J Berkelhamer S Kondapalli J Schumacker P. Sirtuin 3 deficiency does not augment hypoxia-induced pulmonary hypertension. *Am J Respir Cell Mol Biol.* (2013) 49:885–91. 10.1165/rcmb.2013-0191OC 24047466 PMC3931121

[130] Whitaker R Corum D Beeson C Schnellmann R. Mitochondrial biogenesis as a pharmacological target: a new approach to acute and chronic diseases. *Annu Rev Pharmacol Toxicol.* (2016) 56:229–49. 10.1146/annurev-pharmtox-010715-103155 26566156

[131] Hardie DG. AMP-activated/SNF1 protein kinases: conserved guardians of cellular energy. *Nat Rev Mol Cell Biol.* (2007) 8:774–85. 10.1038/nrm2249 17712357

[132] Dodson M Redmann M Rajasekaran N Darley-Usmar V Zhang J. KEAP1-NRF2 signalling and autophagy in protection against oxidative and reductive proteotoxicity. *Biochem J.* (2015) 469:347–55. 10.1042/bj20150568 26205490 PMC5514546

[133] Rainbolt T Saunders J Wiseman R. YME1L degradation reduces mitochondrial proteolytic capacity during oxidative stress. *EMBO Rep.* (2015) 16:97–106. 10.15252/embr.201438976 25433032 PMC4304733

[134] Chen G Kroemer G Kepp O. Mitophagy: an emerging role in aging and age-associated diseases. *Front Cell Dev Biol.* (2020) 8:200. 10.3389/fcell.2020.00200 32274386 PMC7113588

[135] Lemasters J. Selective mitochondrial autophagy, or mitophagy, as a targeted defense against oxidative stress, mitochondrial dysfunction, and aging. *Rejuvenation Res.* (2005) 8:3–5. 10.1089/rej.2005.8.3 15798367

[136] Bakula D Scheibye-Knudsen M. MitophAging: mitophagy in aging and disease. *Front Cell Dev Biol.* (2020) 8:239. 10.3389/fcell.2020.00239 32373609 PMC7179682

[137] He C Klionsky D. Regulation mechanisms and signaling pathways of autophagy. *Annu Rev Genet.* (2009) 43:67–93. 10.1146/annurev-genet-102808-114910 19653858 PMC2831538

[138] Hibshman J Leuthner T Shoben C Mello D Sherwood D Meyer J Nonselective autophagy reduces mitochondrial content during starvation in Caenorhabditis elegans. *Am J Physiol Cell Physiol.* (2018) 315:C781–92. 10.1152/ajpcell.00109.2018 30133321 PMC6336938

[139] Clark I Dodson M Jiang C Cao J Huh J Seol J Drosophila pink1 is required for mitochondrial function and interacts genetically with parkin. *Nature.* (2006) 441:1162–6. 10.1038/nature04779 16672981

[140] Sekine S Youle R. PINK1 import regulation; a fine system to convey mitochondrial stress to the cytosol. *BMC Biol.* (2018) 16:2. 10.1186/s12915-017-0470-7 29325568 PMC5795276

[141] Burman J Pickles S Wang C Sekine S Vargas J Zhang Z Mitochondrial fission facilitates the selective mitophagy of protein aggregates. *J Cell Biol.* (2017) 216:3231–47. 10.1083/jcb.201612106 28893839 PMC5626535

[142] Tanaka A Cleland M Xu S Narendra D Suen D Karbowski M Proteasome and p97 mediate mitophagy and degradation of mitofusins induced by Parkin. *J Cell Biol.* (2010) 191:1367–80. 10.1083/jcb.201007013 21173115 PMC3010068

[143] Twig G Elorza A Molina A Mohamed H Wikstrom J Walzer G Fission and selective fusion govern mitochondrial segregation and elimination by autophagy. *Embo J.* (2008) 27:433–46. 10.1038/sj.emboj.7601963 18200046 PMC2234339

[144] Hoshino A Wang W Wada S McDermott-Roe C Evans C Gosis B The ADP/ATP translocase drives mitophagy independent of nucleotide exchange. *Nature.* (2019) 575:375–9. 10.1038/s41586-019-1667-4 31618756 PMC6858570

[145] Aggarwal S Mannam P Zhang J. Differential regulation of autophagy and mitophagy in pulmonary diseases. *Am J Physiol Lung Cell Mol Physiol.* (2016) 311:L433–52. 10.1152/ajplung.00128.2016 27402690 PMC5504426

[146] Haslip M Dostanic I Huang Y Zhang Y Russell K Jurczak M Endothelial uncoupling protein 2 regulates mitophagy and pulmonary hypertension during intermittent hypoxia. *Arterioscler Thromb Vasc Biol.* (2015) 35:1166–78. 10.1161/atvbaha.114.304865 25814675 PMC4722806

[147] Li B Zhu Y Sun Q Yu C Chen L Tian Y Reversal of the Warburg effect with DCA in PDGF-treated human PASMC is potentiated by pyruvate dehydrogenase kinase-1 inhibition mediated through blocking Akt/GSK-3β signalling. *Int J Mol Med.* (2018) 42:1391–400. 10.3892/ijmm.2018.3745 29956736 PMC6089770

[148] Li T Li S Feng Y Zeng X Dong S Li J Combination of dichloroacetate and atorvastatin regulates excessive proliferation and oxidative stress in pulmonary arterial hypertension development via p38 signaling. *Oxid Med Cell Longev.* (2020) 2020:6973636. 10.1155/2020/6973636 32617141 PMC7306075

[149] Michelakis ED Gurtu V Webster L Barnes G Watson G Howard L Inhibition of pyruvate dehydrogenase kinase improves pulmonary arterial hypertension in genetically susceptible patients. *Sci Transl Med.* (2017) 9:eaao4583. 10.1126/scitranslmed.aao4583 29070699

[150] Wang L Zhang X Cao Y Ma Q Mao X Xu J Mice with a specific deficiency of Pfkfb3 in myeloid cells are protected from hypoxia-induced pulmonary hypertension. *Br J Pharmacol.* (2021) 178:1055–72. 10.1111/bph.15339 33300142

[151] Khan S Cuttica M Beussink-Nelson L Kozyleva A Sanchez C Mkrdichian H Effects of ranolazine on exercise capacity, right ventricular indices, and hemodynamic characteristics in pulmonary arterial hypertension: a pilot study. *Pulm Circ.* (2015) 5:547–56. 10.1086/682427 26401256 PMC4556506

[152] Parra V Bravo-Sagua R Norambuena-Soto I Hernández-Fuentes C Gómez-Contreras A Verdejo H Inhibition of mitochondrial fission prevents hypoxia-induced metabolic shift and cellular proliferation of pulmonary arterial smooth muscle cells. *Biochim Biophys Acta Mol Basis Dis.* (2017) 1863:2891–903. 10.1016/j.bbadis.2017.07.018 28739174

[153] Prins K Thenappan T Weir E Kalra R Pritzker M Archer S. Repurposing medications for treatment of pulmonary arterial hypertension: what’s old is new again. *J Am Heart Assoc.* (2019) 8:e011343. 10.1161/jaha.118.011343 30590974 PMC6405714

[154] Lee D Jung Y. Protective effect of right ventricular mitochondrial damage by cyclosporine a in monocrotaline-induced pulmonary hypertension. *Korean Circ J.* (2018) 48:1135–44. 10.4070/kcj.2018.0061 30403017 PMC6221864

[155] White R Jerjes-Sanchez C Bohns Meyer G Pulido T Sepulveda P Wang K Combination therapy with oral treprostinil for pulmonary arterial hypertension. a double-blind placebo-controlled clinical trial. *Am J Respir Crit Care Med.* (2020) 201:707–17. 10.1164/rccm.201908-1640OC 31765604 PMC7068822

[156] Burki T. Pharmacotherapy for pulmonary arterial hypertension. *Lancet Respir Med.* (2020) 8:e81. 10.1016/s2213-2600(20)30394-5 32857988 PMC7447256

[157] Tuder R Davis L Graham B. Targeting energetic metabolism: a new frontier in the pathogenesis and treatment of pulmonary hypertension. *Am J Respir Crit Care Med.* (2012) 185:260–6. 10.1164/rccm.201108-1536PP 22077069 PMC3297113

[158] Pullamsetti S Savai R Seeger W Goncharova E. Translational advances in the field of pulmonary hypertension. from cancer biology to new pulmonary arterial hypertension therapeutics. Targeting cell growth and proliferation signaling hubs. *Am J Respir Crit Care Med.* (2017) 195:425–37. 10.1164/rccm.201606-1226PP 27627135 PMC5803657

[159] Maihöfer N Suleiman S Dreymüller D Manley P Rossaint R Uhlig S Imatinib relaxes the pulmonary venous bed of guinea pigs. *Respir Res.* (2017) 18:32. 10.1186/s12931-017-0514-0 28178968 PMC5299687

[160] Paulin R Dromparis P Sutendra G Gurtu V Zervopoulos S Bowers L Sirtuin 3 deficiency is associated with inhibited mitochondrial function and pulmonary arterial hypertension in rodents and humans. *Cell Metab.* (2014) 20:827–39. 10.1016/j.cmet.2014.08.011 25284742

[161] Dromparis P Paulin R Sutendra G Qi A Bonnet S Michelakis ED. Uncoupling protein 2 deficiency mimics the effects of hypoxia and endoplasmic reticulum stress on mitochondria and triggers pseudohypoxic pulmonary vascular remodeling and pulmonary hypertension. *Circ Res.* (2013) 113:126–36. 10.1161/circresaha.112.300699 23652801

[162] Diebold I Hennigs J Miyagawa K Li C Nickel N Kaschwich M BMPR2 preserves mitochondrial function and DNA during reoxygenation to promote endothelial cell survival and reverse pulmonary hypertension. *Cell Metab.* (2015) 21:596–608. 10.1016/j.cmet.2015.03.010 25863249 PMC4394191

[163] Zhou H Liu H Porvasnik S Terada N Agarwal A Cheng Y Heme oxygenase-1 mediates the protective effects of rapamycin in monocrotaline-induced pulmonary hypertension. *Lab Invest.* (2006) 86:62–71. 10.1038/labinvest.3700361 16357868

[164] Zhao Q Song P Zou MH. AMPK and pulmonary hypertension: crossroads between vasoconstriction and vascular remodeling. *Front Cell Dev Biol.* (2021) 9:691585. 10.3389/fcell.2021.691585 34169079 PMC8217619

[165] Pak O Scheibe S Esfandiary A Gierhardt M Sydykov A Logan A Impact of the mitochondria-targeted antioxidant MitoQ on hypoxia-induced pulmonary hypertension. *Eur Respir J.* (2018) 51:1701024. 10.1183/13993003.01024-2017 29419444

[166] Hsu C Roan J Fang S Chiu M Cheng T Huang C Transplantation of viable mitochondria improves right ventricular performance and pulmonary artery remodeling in rats with pulmonary arterial hypertension. *J Thorac Cardiovasc Surg.* (2022) 163:e361–73. 10.1016/j.jtcvs.2020.08.014 32948302

[167] Thompson A Lawrie A. Targeting vascular remodeling to treat pulmonary arterial hypertension. *Trends Mol Med.* (2017) 23:31–45. 10.1016/j.molmed.2016.11.005 27989641

[168] Park A Tran D Schaal J Wang M Selsted M Beringer P. Preclinical pharmacokinetics and safety of intravenous RTD-1. *Antimicrob Agents Chemother.* (2022) 66:e0212521. 10.1128/aac.02125-21 35041507 PMC8923172

